# Correction: The PROGRAM study: awake mapping versus asleep mapping versus no mapping for high-grade glioma resections: study protocol for an international multicenter prospective three-arm cohort study

**DOI:** 10.1136/bmjopen-2020-047306corr1

**Published:** 2022-08-08

Gerritsen JKW, Dirven CMF, De Vleeschouwer S, *et al*. The PROGRAM study: awake mapping versus asleep mapping versus no mapping for high-grade glioma resections: study protocol for an international multicenter prospective three-arm cohort study. *BMJ Open* 2021;11:e047306. doi: 10.1136/bmjopen-2020-047306

The authors want to notify the readers on the updates done in the published version. We have added two authors to the author list: neuro-linguistic expert Dr Djaina D Satoer (Department of Neurosurgery, Erasmus MC, Rotterdam, The Netherlands), who has been of tremendous in developing the neuro-linguistic test battery for the PROGRAM study; and neurosurgeon and neurophysiologist Dr Kathleen Seidel (Department of Neurosurgery, Inselspital Universitätsspital Bern, Bern, Switzerland), who has co-developed the Bern protocol for asleep motor mapping. This specific mapping protocol for the Bern location has been added to the revised manuscript.

Moreover, we have added “Object Naming” to the neuro-linguistic test battery and more extensive quality of life questionnaires (EORTC QLQ-C30, EORTC QLQ-BN20) and MOCA to the data collection.

Last, the registration of the use and the potential effect of preoperative steroids and the integrity of the subcortical tracts on DTI has been added.

