# Supplementary material for: The PROGRAM study: awake mapping versus asleep mapping versus no mapping for high-grade glioma resections: study protocol for an international multicenter prospective three-arm cohort study
Source: BMJ Open. 2021 Jul 21;11(7):e047306. doi: 10.1136/bmjopen-2020-047306 (PMC8296818; doi:10.1136/bmjopen-2020-047306)
Supplement: Author's manuscript [file bmjopen-2020-047306.draft_revisions.pdf]

BMJ Open

BMJ Open is committed to open peer review. As part of this commitment we make the peer review history of every article we publish publicly available.

When an article is published we post the peer reviewers' comments and the authors' responses online. We also post the versions of the paper that were used during peer review. These are the versions that the peer review comments apply to.

The versions of the paper that follow are the versions that were submitted during the peer review process. They are not the versions of record or the final published versions. They should not be cited or distributed as the published version of this manuscript.

BMJ Open is an open access journal and the full, final, typeset and author-corrected version of record of the manuscript is available on our site with no access controls, subscription charges or pay-per-view fees (http://bmjopen.bmj.com).

If you have any questions on BMJ Open's open peer review process please email info.bmjopen@bmj.com

BMJ Open

The PROGRAM-study: Awake Mapping versus Asleep Mapping versus No Mapping for High-Grade Glioma Resections: Study Protocol for An International Multicenter Prospective 3-Arm Cohort Study

Journal:	*BMJ Open*
Manuscript ID	bmjopen-2020-047306
Article Type:	Protocol
Date Submitted by the Author:	24-Nov-2020

Complete List of Authors:	Gerritsen, Jasper; Erasmus MC, Department of Neurosurgery<br>Dirven, Clemens; Erasmus MC, Department of Neurosurgery<br>De Vleeschouwer, Steven; Katholieke Universiteit Leuven UZ Leuven, Department of Neurosurgery<br>Schucht, Philippe; Inselspital Universitätsspital Bern, Department of Neurosurgery<br>Berger, Mitchel; University of California San Francisco, Department of Neurosurgery<br>Broekman, Marike; Medisch Centrum Haaglanden, Department of Neurosurgery<br>Vincent, Arnaud; Erasmus MC, Department of Neurosurgery

Keywords:	NEUROSURGERY, SURGERY, Neurological oncology < NEUROLOGY, Neurosurgery < SURGERY

SCHOLARONE™ Manuscripts

I, the Submitting Author has the right to grant and does grant on behalf of all authors of the Work (as defined in the below author licence), an exclusive licence and/or a non-exclusive licence for contributions from authors who are: i) UK Crown employees; ii) where BMJ has agreed a CC-BY licence shall apply, and/or iii) in accordance with the terms applicable for US Federal Government officers or employees acting as part of their official duties; on a worldwide, perpetual, irrevocable, royalty-free basis to BMJ Publishing Group Ltd ("BMJ") its licensees and where the relevant Journal is co-owned by BMJ to the co-owners of the Journal, to publish the Work in this journal and any other BMJ products and to exploit all rights, as set out in our licence.

The Submitting Author accepts and understands that any supply made under these terms is made by BMJ to the Submitting Author unless you are acting as an employee on behalf of your employer or a postgraduate student of an affiliated institution which is paying any applicable article publishing charge ("APC") for Open Access articles. Where the Submitting Author wishes to make the Work available on an Open Access basis (and intends to pay the relevant APC), the terms of reuse of such Open Access shall be governed by a Creative Commons licence – details of these licences and which Creative Commons licence will apply to this Work are set out in our licence referred to above.

Other than as permitted in any relevant BMJ Author's Self Archiving Policies, I confirm this Work has not been accepted for publication elsewhere, is not being considered for publication elsewhere and does not duplicate material already published. I confirm all authors consent to publication of this Work and authorise the granting of this licence.

For peer review only

The PROGRAM-study: Awake Mapping versus Asleep Mapping versus No Mapping for High-Grade Glioma Resections: Study Protocol for An International Multicenter Prospective 3-Arm Cohorts Study.

Running head: The PROGRAM-study

Jasper K.W. Gerritsen[1] MD (Corresponding author)
Clemens M.F. Dirven[1] MD PhD
Steven de Vleeschouwer[2] MD PhD
Philippe Schucht[3] MD PhD
Mitchel Stuart Berger[5] MD PhD
Marike L.D. Broekman[6] MD PhD
Arnaud J.P.E. Vincent[1] MD PhD

[1]Erasmus Medical Center Rotterdam, The Netherlands, Department of Neurosurgery
[2]University Hospital Leuven, Belgium, Department of Neurosurgery
[3]Inselspital, Universitätsspital Bern, Switzerland, Department of Neurosurgery
[4]University of California, San Francisco, United States, Department of Neurosurgery
[5]Haaglanden Medisch Centrum Den Haag, The Netherlands, Department of Neurosurgery

Corresponding author
Name: Jasper K.W. Gerritsen MD
Address: Dr. Molewaterplein 40, 3015 GD Rotterdam, The Netherlands
Email: j.gerritsen@erasmusmc.nl

Keywords: Glioblastoma – Neurological morbidity – Extent of resection – Survival
Word count: 4613
Funding: none
NTR: NL8998
Ethics Committee: Issuing body: Erasmus MC, ID: MEC-2020-0812
Version 3.0 14-09-2020

For peer review only - http://bmjopen.bmj.com/site/about/guidelines.xhtml

ABSTRACT

Introduction

The main surgical dilemma during glioma resections is the surgeon's inability to accurately identify eloquent areas when the patient is under general anesthesia (GA) without mapping techniques. Intraoperative stimulation mapping (ISM) techniques can be used to maximize extent of resection in eloquent areas yet simultaneously minimze the risk of postoperative neurological deficits. ISM has been widely implemented for low-grade glioma resections (LGG) backed with ample scientific evidence, but this is not yet the case for high-grade glioma (HGG) resections. Therefore, ISM could thus be of important value in HGG surgery to improve both surgical and clinical outcomes.

Methods and Analysis

This study is a international, multicenter, prospective 3-arm cohort study of observational nature. Consecutive HGG patients will be operated with awake mapping, asleep mapping or no mppaing with a 1:1:1 ratio.

Primary endpoints are: 1) Proportion of patients with NIHSS (National Institute of Health Stroke Scale) deterioration at 6 weeks, 3 months and 6 months after surgery and 2) Extent of resection as assessed by a neuroradiologist on postoperative contrast MRI scans. Secondary endpoints are: 1) Overall survival (OS); 2) Progression-free survival (PFS) at 6 months and 12 months after surgery; 3) Onco-functional outcome and 4) Frequency and severity of Serious Adverse Events (SAEs) in each arm. Total duration of the study is 5 years. Patient inclusion is 4 years, follow-up is 1 year.

Ethics and Dissemination

The study has been approved by the Medical Ethics Committee (METC Zuid-West Holland/Erasmus Medical Center; MEC-2020-0812). The results will be published in peer-reviewed academic journals and disseminated to patient organisations and media.

Strenghts and limitations

- First multicenter prospective study directly comparing awake mapping, asleep mapping and no mapping for glioblastoma resections in or near eloquent areas.
- International, multicenter design on a large scale, which will help generalize the results to more centers and countries.
- Observational design will not exclude all possible, inherent forms of bias.

For peer review only

INTRODUCTION

Gliomas represent the most frequent class of malignant tumors of the central nervous system (CNS)[1,2]. The World Health Organization (WHO) classifies them into grades 1-4, where grade 1 and -2 consist of low-grade gliomas (LGG) and grades 3 and -4 represent high-grade gliomas (HGG)[1,2]. Grade 4 gliomas are also more commonly referred to as glioblastoma multiforme (GBM). Gliomas, and in particular high-grade gliomas such as GBMs, are relatively rare (incidence of 5/100,000 persons/year in Europe and North America), but are associated with a relatively high morbidity and mortality regardless of years of scientific efforts to improve clinical outcomes in these patients[1-4]. As to date, the median survival for GBMs is 12-15 months and no curative therapy is available[1-4].

Studies show that maximizing the extent of resection results in improved patient survival rates[8-15]. Moreover, patients with gross-total resections (GTR) derived the most benefit from the adjuvant chemoradiotherapy compared to patient with subtotal resections[16]. Though, in excess of 50% of gliomas are located in- or near eloquent areas of the brain[2]. Eloquent areas are important areas within the brain where speech and/or motor functions are located. Damaging these areas during surgery can lead to severe and permanent neurological deficits that seriously impact the quality of life. As a consequence of this worsened condition, some patients are excluded for radio- and chemotherapy, leading to suboptimal clinical outcomes[16].

Thus, the main surgical problem for the surgeon is the inability to accurately identify these eloquent areas when the patient is under general anesthesia (GA) when no brain mapping techniques are being used. Therefore, when resecting gliomas in these areas, they are not always operated as aggressive as possible, due to the chance of seriously damaging the patient with a rather low life expectancy[2,10,12-15]. Therefore, a surgical technique optimizing resection of the tumor in eloquent areas yet simultaneously preventing neurological deficits is necessary to improve extent of resection and survival while preserving quality of life and neurological functioning in these patients.

Intraoperative stimulation mapping (ISM) is the term for surgical techniques used during glioma resection to map eloquent brain areas by stimulating specific (sub)cortical areas intraoperatively[17]. Awake craniotomy (AC) is among the most widespread mapping techniques used during glioma resection. During an awake craniotomy, the patient is awake

For peer review only

and cooperative during the resection of the tumor while the surgeon uses electro(sub)cortical mapping to prevent damage to eloquent areas during resection[18].

The use of ISM techniques has tremendous potential in glioma resections in eloquent areas, not only for LGG but for HGG as well. However, no international consensus has been reached regarding the use of these techniques in HGG patients. The scientific evidence for the use of these techniques in this patient group is currently both inconclusive and fragmented. Therefore, we propose an international, multicenter prospective clinical trial, in which the use of ISM techniques in HGG patients will be evaluated.

The described research initiative will be able to study these techniques in a prospective setting while covering a breath of centers and countries. Hence, the data generated in this research collaboration will be able to answer multiple research questions with excellent generalizability, external validity and overall quality in both a cost-effective and practical setting.

For peer review only - http://bmjopen.bmj.com/site/about/guidelines.xhtml

METHODS/DESIGN

Study design

This is a international, multicenter, prospective, 3-arm cohort study. Eligible patients are operated using awake mapping, asleep mapping or no mapping with a 1:1:1 ratio with a sequential computer-generated random number as subject ID.

Study objectives

The primary study objective is to increase safety and efficacy during surgery (by decreasing neurological morbidity) and to optimize extent of resection (EOR) in GBM patients as expressed by NIHSS scores and volumetric data. Secondary study objectives are to improve overall survival (OS), progressive-free survival (PFS) and onco-functional outcome as expressed by survival data, progression on MRI scans and combining postoperative EOR/NIHSS outcomes respectively.

Study setting and participants

Patients will be recruited for the study from the neurosurgical or neurological outpatient clinic or through referral from general hospitals of the participating neurosurgical hospitals, located in Europe and the United States. The study is open to additional participating neurosurgical centers.

Patient and public involvement statement

Patients enrolled in the SAFE-trial (awake craniotomy versus craniotomy under general anesthesia for glioblastoma patients, NCT03861299) were consulted for this study to include patient experiences with resections with- and without mapping.

Inclusion criteria

In order to be eligible to participate in this study, a subject must meet all of the following criteria:

1. Age ≥18 years and ≤ 90 years
2. Tumor diagnosed as HGG on MRI as assessed by the neurosurgeon
3. Tumors situated in or near eloquent areas; motor cortex, sensory cortex, subcortical pyramidal tract, speech areas or visual areas as indicated on MRI (Sawaya Grading II and II)[38]

For peer review only - http://bmjopen.bmj.com/site/about/guidelines.xhtml

4. The tumor is suitable for resection (according to neurosurgeon)
5. Written informed consent

Exclusion criteria

A potential subject who meets any of the following criteria will be excluded from participation in this study:

1. Tumors of the cerebellum, brain stem or midline
2. Multifocal contrast enhancing lesions
3. Medical reasons precluding MRI (e.g. pacemaker)
4. Inability to give written informed consent (e.g. because of severe language barrier)
5. Secondary high-grade glioma due to malignant transformation from low-grade glioma
6. Second primary malignancy within the past 5 years with the exception of adequately treated in situ carcinoma of any organ or basal cell carcinoma of the skin

Participant timeline

The flow diagram (Figure 1) displays the main study procedures, including follow-up evaluations. In summary, study patients will be evaluated at presentation (baseline), during their hospital stay, at discharge and during the follow-up period at 6 weeks, 3 months, 6 months and 12 months postoperatively. Preoperatively, neurological morbidity will be evaluated using the NIHSS and MRC scale. After these baseline assessments, patients will be allocated to either the awake mapping, asleep mapping or no mapping group. At 6 weeks, 3 months and 6 months postoperatively, neurological morbidity and oncofunctional outcome will be evaluated as part of patient follow-up. Overall survival and progression-free survival will be assessed at 12 months postoperatively. We expect to complete patient inclusion in 4 years. The estimated duration of the study (including follow-up) will be 5 years.

Interventions

(1) Awake craniotomy with local anesthesia (arm 1: awake mapping).

On the evening before surgery 1.5–2.0 mg lorazepam is administered for anxiolysis. Supplemental O_2 might be provided through a nasal canular. The patient is sedated with a bolus injection of propofol (0.5–1 $mg.kg^{-1}$) and kept sedated with a propofol infusion pump

For peer review only - http://bmjopen.bmj.com/site/about/guidelines.xhtml

(mean: 4 $mg.kg^{-1}.h^{-1}$) and remifentanil ((0.5-2 µg/kg/min). An arterial line (with standard monitoring for vital signs in addition to BP monitoring), central venous catheter, and urinary catheter are inserted. The patient is awakened and positioned on the table. At this point local anaesthesia for the fixation of the head in the Mayfield clamp and the surgical field is provided with a mixture of 10 mL lidocaine 2% with 10 mL bupivacaine 0.5% plus adrenaline 1:200,000 for the Mayfield clamp and up to 40 mL bupivacaine 0.375% with adrenaline 1:200,000 for the surgical field.

After positioning, clamp fixation, and surgical field infiltration, patients are sedated again for the trephination until the dura mater is opened, after local application of some drops of local anaesthetics. Propofol sedation is stopped after opening of the dura, with the patient awakening with as few external stimuli as possible. Cortical stimulation is performed with a bipolar electrical stimulator. The distance between both poles is 5 mm, and stimulation is performed by placing this bipolar pincet directly on the cortical surface and stimulating with increasing electrical biphasic currents of 2–12 mA (pulse frequency 60 Hz, single pulse phase duration of 100 microsec.) until motor or speech arrest is observed. For motor mapping a 2-second train and for speech mapping a 5-second train is used, respectively.

The Boston naming test and repetition of words is done in cooperation with a neuropsychologist/linguist, who will inform the neurosurgeon of any kind of speech arrest or dysarthria. The difference between these is not always clear, but can be distinguished from involuntary muscle contraction affecting speech. When localizing the motor and sensory cortex, the patient is asked to report any unintended movement or sensation in extremities or face.

Functional cortical areas are marked with a number. After completion of cortical mapping, a resection of the tumour is performed as radical as possible using an ultrasonic aspirator and suction tube, while sparing these functional areas. When the tumour margins or white matter is encountered or when on regular neuronavigation the eloquent white matter tracts are thought to be in close proximity, subcortical stimulation (biphasic currents of 8–16 mA, pulse frequency 60 Hz, single pulse phase duration of 100 microsec., 2-second train) is performed to localize functional tracts. If subcortical tracts are identified, resection is stopped. During the resection of the lesion close to an eloquent area, the patient is involved in a continuous dialogue with the neuropsychologist. That way the neurosurgeon has 'online'-control of these eloquent areas. In case of beginning disturbances of communication or of motor or sensory sensations the resection is cessated immediately. When, due to stimulation, an epileptic seizure occurs, this is stopped by administering some drops of iced saline on the just

For peer review only - http://bmjopen.bmj.com/site/about/guidelines.xhtml

stimulated cortical area. Continuous corticography may be used to monitor after discharge potentials to identify subclinical seizure activity. If a seizure continues, an i.v. propofol bolus of 0.5 mg/kg is administrated and repeated until the seizure stops. After resection of the tumour a final neurological examination is performed. During closure of the surgical field the patient is sedated with propofol again. After wound closure and dressing, sedation is stopped. The awake patient is transferred to the post-anaesthesia care unit, where the patient is hemodynamically and neurologically monitored for 24 hours.

(2) Asleep mapping under general anesthesia (arm 2: asleep mapping).

An IV is started on ipsilateral hand to the tumor. Premedicate with up to 2 mg of midazolam. None if altered mental status (prevent further increase in ICP). Arterial (ipsilateral to tumor) catheter is inserted after induction of anesthesia. Anesthesia goals are to decrease ICP (if high), to maintain adequate CPP (at least 70 mmHg) to prevent cerebral ischemia from brain retraction, and to allow intraoperative cortical motor mapping. Patients typically receive 1-2 g of cefazolin, and 4 mg of decadron before skin incision, and sometimes up to 1 g/kg of mannitol (verify all with surgeon). Keep the room warm and patient covered as the goal is to have the core temperature above 36 C° during motor mapping. Induction with propofol. In case of increased ICP, have patient hyperventilate during preoxygenation and continue hyperventilation with mask as soon as possible after induction of anesthesia. Fentanyl up to 5 µg/kg in divided doses throughout induction, prior to intubation. Verify adequate neuromuscular blockade (rocuronium) prior to intubation to avoid coughing/straining. Tape eyes, and insert at least one additional large bore IV (don't use the contralateral hand/arm). Then let neuromuscular relaxation wear off for motor mapping (do not reverse). Patient position will depend on location of tumor. Maintain anesthesia with 70% nitrous oxide in oxygen, low dose inhalation agent (less than 0.5 MAC), and a remifentanil (0.2 µg/kg/min) or fentanyl infusion (2 µg/kg/hr). Maintain euvolemia (Lactated Ringer's). Use mild hyperventilation (PaCO2 35 mmHg). Once the bone flap is removed, have the surgeon assess the tightness of the dura. Decrease ICP further if necessary (pCO2, mannitol, propofol, head up etc.). Once the dura is open, the goal is to avoid "brain shift" so that stereotactic navigation system can be used optimally. During motor mapping, have the arm, leg and face uncovered to observe for movement. Stimulation is performed with the use of evoked potentials and continuous dynamic mapping/direct subcortical stimulation (CDM/DSS) with a monopolar stimulator (INOMED© Medizintechnik GmBH, Germany). During stimulation,

For peer review only

TES-MEP registration is performed of the contralateral m. orbicularis oris, m. orbicularis oculi, m. biceps brachii, m. abductor pollicis, m. rectus femoris and m. tibialis anterior; and the ipsilateral m. abductor pollicis. SSEP registration is performed of the contralateral n. tibialis and bilateral n. medianus. The pulse form is negative, with 5 pulses and a pulse width of 500 μs, ISI 4 and current between 5-20 mA. In case of poststimulation continuation of motor activity, surgeon will try to stop it by applying cold saline on the cortex. Have propofol (10 mg/ml) in line in case of intraoperative seizures (0.5 mg/kg for seizure suppression). May use neuromuscular relaxants after the last motor mapping. Fentanyl infusion is usually stopped at the beginning of closure. Remifentanil infusion is stopped about 10 min before end of surgery. At this point, use of inhalation agent may be replaced with a propofol infusion (50-100 μg/kg/min). Normalize pCO_2 to facilitate spontaneous breathing at the end of the operation. Use of inhalation agents (or propofol) is usually stopped about 10-15 min before end of surgery, and nitrous oxide at the end of surgery. Reverse residual neuromuscular blockade once the Mayfield pins have been removed. At the end of the procedure all anaesthetics are stopped and patient is brought to the Post Anaesthesia Care Unit (PACU/IC). Detubation of the patient is performed as early as possible, if patient fulfils the detubation criteria (> 36 C body temperature, stable hemodynamics, sufficient spontaneous ventilation, adequate response to verbal orders). Postoperative analgesia is provided with Paracetamol i.v. or p.o. 1 g up to 4 dd and morphine 7.5 mg s.c. up to 4 dd, if necessary. At the PACU the patient is hemodynamically and neurologically monitored for 24 hours.

(3) Craniotomy under general anaesthesia without mapping (arm 3: no mapping).

On the evening before surgery 1.5–2.0 mg lorazepam is administered for anxiolysis. 60 min. before anaesthesia induction the patient receives 1g paracetamol p.o. and 7.5-15 mg midazolam p.o. if requested for sedation. En route to the operating room, 0.5-2 mg midazolam i.v. may be given. 1g cefazoline is given iv. for antibiotic prophylaxis before anaesthesia induction. General anaesthesia is induced intravenously with fentanyl 0.25-0.5 mg, propofol 100-200 mg and cis-atracurium 10-20 mg. After induction of anaesthesia, patient is orotracheally intubated and mechanical ventilation is applied. Respiratory rate and tidal volume are adjusted to keep the patient normocapnic.

An arterial line (alternatively: two peripheral i.v.'s), central venous catheter (v. basilica), and urinary catheter are inserted. Anaesthesia is maintained with propofol (up to 10 mg/kg/h) and remifentanil (0.5-2 μg/kg/min). isoflurane (up to 1 MAC) and clonidine (1-2 μg/kg) may be

For peer review only - http://bmjopen.bmj.com/site/about/guidelines.xhtml

added for maintenance, if necessary (a beta blocker or calcium channel blocker may be used to control BP as an alternative to clonidine). The fluid management is aiming for normovolemia. 0.9% saline solution and balanced crystalloids are used for maintenance, in case of blood loss > 300 ml, HAES 130/0.4 solution will be given.

Temperature management is aiming for normothermia, warm-air blankets and warmed infusion lines are used. Arterial blood gas analysis is performed at the beginning of the procedure and repeated, if necessary. Electrolytes are controlled and substituted and hyperglycemia will be treated with insulin, if necessary.

The anesthetized patient is positioned on the table. Local infiltration of the scalp is performed with 20 ml lidocaine 1% with adrenaline 1:200.000 to reduce bleeding. The insertion points of the Mayfield clamp are not infiltrated with local anaesthetics.

Trephination and tumour resection are performed without any additional neuro-psychological monitoring, guided by BRAINLAB-neuronavigation. At the end of the procedure all anaesthetics are stopped and patient is brought to the Post Anaesthesia Care Unit (PACU/IC). Detubation of the patient is performed as early as possible, if patient fulfils the detubation criteria (> 36 C body temperature, stable hemodynamics, sufficient spontaneous ventilation, adequate response to verbal orders). Postoperative analgesia is provided with paracetamol i.v. or p.o. 1 g up to 4 dd and morphine 7.5 mg s.c. up to 4 dd, if necessary. At the PACU the patient is hemodynamically and neurologically monitored for 24 hours.

Intraoperative Imaging:

The use of fMRI, DTI (Diffusion Tensor Imaging), ultrasound or 5-ALA is allowed to be used in both groups on the surgeons indication.

<u>Outcomes</u>

Primary outcome measures

The primary outcomes are 1) proportion of patients with NIHSS (National Institute of Health Stroke Scale) deterioration at 6 weeks, 3 months and 6 months postoperatively, in which deterioration is defined as an increase of at least one point on the total NIHSS score compared to this score at baseline and 2) extent of resection (EOR) as assessed by a neuroradiologist on postoperative contrast MRI scans using volumetric analyses;

Secondary outcome measures

For peer review only

The secondary outcomes are 1) progression-free survival (PFS) at 12 months defined as time from diagnosis to disease progression (occurrence of a new tumor lesions with a volume greater than 0.175 cm^3, or an increase in residual tumor volume of more than 25%) or death, whichever comes first; 2) overall survival (OS) at 12 months defined as time from diagnosis to death from any cause; 3) onco-functional outcome defined as the calculated coordinate of the EOR on the x-axis and the postoperative NIHSS deterioration on the y-axis and 4) Frequency and severity of Serious Adverse Events (SAEs) in each arm.

NIHSS

The National Institutes of Health Stroke Scale, or NIH Stroke Scale (NIHSS) is a tool used by healthcare providers to objectively quantify the impairment caused by a stroke, but has been used extensively for outcome in glioma surgery because of the lack of such scale for neuro-oncologic purposes and has been validated. The NIHSS is composed of 11 items, each of which scores a specific ability between a 0 and 4. For each item, a score of 0 typically indicates normal function in that specific ability, while a higher score is indicative of some level of impairment. The individual scores from each item are summed in order to calculate a patient's total NIHSS score. The maximum possible score is 42 and the minimum score 0.

For peer review only

<u>Study procedures: Clinical evaluations and follow-up</u>

1. Baseline

1) Assessment of baseline symptom(s) and medical history
2) Full neurological examination (NIHSS, MRC)

2. Preoperatively

1) MRI-brain-navigation with Gd-contrast (standard procedure)

3. Postoperatively

1) MRI-brain with Gd-contrast within <72 hours postoperatively
    a. EOR volumetric assessment by two independent neuroradiologists
2) Description of presenting symptom(s) at day 1-2-3- postoperatively
3) Full neurological examination at day 1-2-3 postoperatively

4. 6 weeks follow up after surgery

1) Description of presenting symptom(s)

For peer review only - http://bmjopen.bmj.com/site/about/guidelines.xhtml

2) Full neurological examination (NIHSS, MRC), onco-functional outcome

5. 3 months follow up after surgery

1) Description of presenting symptom(s)
2) Full neurological examination (NIHSS, MRC), onco-functional outcome
3) MRI-brain with Gd-contrast

6. 6 months follow up after surgery

1) Description of presenting symptom(s)
2) Full neurological examination (NIHSS, MRC), onco-functional outcome
3) MRI-brain with Gd-contrast

7. 12 months follow up after surgery

1) Overall survival (as assessed by digital medical records of the hospital)
2) Progression-free survival (as assessed by routine MRI)

Sample size

This study has *two* primary endpoints. In order to guarantee that the overall type I error rate does not exceed 5%, we apply a weighted Bonferroni correction for multiple testing. The sample size calculations that follow take that into account.

For the first primary endpoint, proportion of patients with neurological deterioration at 6 weeks post- surgery, we assume a deterioration rate of 10% in the control group, and 3% in the experimental group.

A two-sample test for proportions with continuity correction requires 411 patients (137 per arm) in total in order to detect the above-mentioned difference of 7% with 80% power at a 4% significance level.

For the second primary endpoint, proportion of patients without residual contrast-enhancing tumor on postoperative MRI, we assume a success rate of 25% in the control group, and 50% in the experimental group. A two-sample test for proportions with continuity correction requires 188 patients (94 per arm) in total in order to detect the above-mentioned difference of 25% with 80% power at a 1% significance level.

For peer review only - http://bmjopen.bmj.com/site/about/guidelines.xhtml

In order to power the study for both primary endpoints, we should include the larger required number of patients, i.e. 411. A total of 411 eligible and evaluable patients in three arms allow the difference of 25% in proportion of patients without residual tumor to be detected with 88% power. Taking into account possible ineligibility and withdrawal of consent (we estimate this at 10%), a total of 453 patients will be included (151 patients per arm).

<u>Data collection</u>

All patient data is collected in the electronic data software Castor EDC. This software allows built-in logical checks and validations to promote data quality. Data entry and group allocation is performed by the study coordinator or locally by trained physicians and research nurses under supervision of the local investigator.

<u>Data analysis</u>

All analyses will be according the intention to treat principle, restricted to eligible patients. Patients initially registered but considered ineligible afterwards based on the histological analysis on tissue extracted during surgery, will be excluded from all analyses.

Primary study parameter(s)

The primary endpoints will be analyzed using multivariate logistic regression, where treatment group effect will be corrected for minimization factors age group (≤55 years vs >55years), Karnofsky performance scale (80–90 vs >90), and left or right hemisphere (presented in order of decreasing prognostic value).

As the frequency of neurological deterioration is expected to be relatively low, we may not be able to correct for all stratification factors as mentioned above. We will be including a stratification factor in the primary analysis model with each 10 observed events using the order of prognostic value as mentioned in the paragraph above, where the first 10 events will be used to estimate the effect of the arm. This rule will be applied in case less than 40 patients in total develop neurological deterioration. In the so constructed multivariate logistic regression model the treatment arm effect will be tested at 4% significance level. The primary analysis of proportion of patients without residual contrast-enhancing tumour consist of a multivariate logistic regression, where arm effect is corrected for all minimization factors. In this model the group effect will be tested at 1% significance level. Manual or semiautomatic segmentation will be performed on axial T1 MRI contrast enhanced slices to measure tumor volume. A determination of volumes will be calculated blinded for the treatment group.

For peer review only - http://bmjopen.bmj.com/site/about/guidelines.xhtml

Secondary study parameters

The Kaplan-Meier method will be used to estimate PFS and OS proportions per treatment group at appropriate time points, while the Greenwood estimate of the standard error will be used to construct the corresponding 95% CI. Multivariate Cox proportional hazards models will be built for PFS and OS where treatment group effect will be corrected for minization factors age group (≤55 years vs >55years), Karnofsky performance scale (80–90 vs >90), and left or right hemisphere. Additionally, competing risk analysis will be used to calculate cumulative incidence of PFS (with competing risks progression/relapse and death without progression/relapse which add up to 100% at every time point). SAE's in both groups will be described.

Study monitoring

No scheduled on-site monitoring visits will be performed. Local investigators will remain responsible for the fact that the rights and well-being of patients are protected, the reported trial data are accurate, complete, and verifiable from source documents and the conduct of the trial is in compliance with the currently approved protocol/amendment(s), with GCP, and with the applicable regulatory requirement(s). Direct access to source documentation (medical records) must be allowed for the purpose of verifying that the data recorded in the CRF are consistent with the original source data. No Data Safety Monitoring Board will be installed: all interventions are care-as-usual and patients are allocated without randomisation.

Adverse events (AEs) and serious adverse events (SAEs)

Adverse events are defined as any undesirable experience occurring to a subject during the study, whether or not considered related to neurosurgery. All adverse events reported spontaneously by the subject or observed by the investigator or his staff will be recorded from start of surgery until 6 weeks after surgery. Serious adverse events are any untoward medical occurrence or effect that results in death; is life-threatening (at the time of the event); requires hospitalization or prolongation of existing inpatients' hospitalization; results in persistent or significant disability or incapacity or any other important medical event that did not result in any of the outcomes listed above due to medical or surgical intervention, but could have been based upon appropriate judgement by the investigator. An elective hospital admission will not be considered as a serious adverse event. Most of the (serious) adverse effects of treatments (awake surgery or surgery under generalised anaesthesia) will be mainly related to the surgery: post operative pain, nausea and anaemia (in case of massive blood

For peer review only - http://bmjopen.bmj.com/site/about/guidelines.xhtml

loss), Infections, intracranial haemorrhage, epilepsy, aphasia, paresis/paralysis in arms or/and legs.

Most of the (serious) adverse effects of treatments (awake surgery or surgery under generalised anaesthesia) will be mainly related to the surgery: postoperative pain, nausea and anaemia (in case of massive blood loss), infections, intracranial haemorrhage, epilepsy, aphasia, paresis/paralysis in arms or/and legs. The neurological morbidity is under investigation in this trial and well-known risk / complications of the craniotomy and can be attributed to the nature of the operation. Neurosurgical clinics are well adapted to prevent and treat such events. SAEs will be collected through routine data management.

<u>Publication of results</u>

Trial results will be published in an international journal, communicated to neurological and neurosurgical associations and presented at (inter)national congresses.

For peer review only

DISCUSSION

During glioblastoma resection, neurosurgeons face a dilemma: maximizing extent of resection while minimizing risk of postoperative neurological complications (morbidity, deficits). The use of ISM techniques such as awake mapping (awake craniotomy, AC) and asleep mapping equip the surgeon intraoperatively with the needed information to balance these two surgical goals.

There is ample scientific evidence that AC increases resection percentage while preserving quality of life (QoL) in low-grade glioma (LGG) patients[19-30]. However, only very few studies have reported the use of AC in GBM, while AC could potentially be of important value in GBM surgery as well[17,19, 21-23,30]. Recent (retrospective) evidence shows that patients with GBM operated with AC had significant less postoperative neurological morbidity and significantly higher percentage of total resections[31,32]. These results were in line with (among others) the landmark paper of De Witt Hamer et al, in which they concluded that ISM/AC improves clinical outcomes for glioma patients (both LGG and HGG), most noticeably a decreased incidence of late major neurological deficits[17]. Thus, solid prospective evidence of the use of ISM in GBM patients is vital to strengthen the scientific evidence for the use of this techniques in this patient group.

Besides from “awake” brain mapping (with AC), “asleep” mapping methods are another excellent ISM tool to preserve these tracts (MEP, SSEP, continuous dynamic mapping)[33-35]. For example, the neurosurgical department at the Inselspital, Universitätsspital (Bern, Switzerland) uses continuous dynamic mapping with monopolar stimulation for glioma surgeries adjacent to motor eloquent areas[34-35]. They realize continuous (temporal coverage) and dynamic (spatial coverage) mapping by integrating the mapping probe at the tip of the suction device where acoustic feedback indicates proximity to the corticospinal tract. New intraoperative developments like these can be combined with ever-improving diagnostics and radiomics (DTI, HARDI q-ball imaging) to yield optimal results in glioma surgeries in eloquent areas[36,37].

ISM techniques, whether awake or asleep, have been thoroughly demonstrated as an effective surgical technique in the current literature for low-grade glioma. ISM is showing promising results as a technique used for glioblastoma resections, in particular in eloquent areas. This

For peer review only - http://bmjopen.bmj.com/site/about/guidelines.xhtml

study aims to evaluate whether the use of ISM is the appropriate answer to the surgical dilemma of maximizing EOR while minimzing neurological morbidity and furthremore, to identify if a specific form of ISM yields superior clinical outcomes in subgroups of patients.

Trial status

The study will start at February 1st, 2021 and is open to additional participating neurosurgical centers.

Figure 1: Study flowchart

For peer review only

ABBREVIATIONS

5-ALA: 5-Aminolevulinic Acid

AC: Awake Craniotomy

AE: Adverse Event

CI: Confidence Interval

CTC: Clinical Trial Center

DTI: Diffusion Tensor Imaging

EMC: Erasmus Medical Center

EOR: Extent of Resection

fMRI: Functional Magnetic Resonance Imaging

GA: General Anesthesia

GCP: Good Clinical Practice

GTR: Gross-Total Resection

HMC: Haaglanden Medical Center

HRQoL: Health-related Quality of Life

ISM: Intraoperative Stimulation Mapping

KPS: Karnofsky Performance Score

LGG: Low-grade glioma

METC: Medical Ethics Committee

NIHSS: National Institute of Health Stroke Scale

OS: Overall survival

PACU: Post-Anesthesia Care Unit

PFS: Progression-free survival

SAE: Serious Adverse Event

STR: Subtotal Resection

UCSF: Univeristy of California, San Francisco

WHO: World Health Organization

For peer review only

DECLARATIONS

Author contributions

JG, AV and MLB designed the study, wrote the study protocol and are end-responsible for the implementation and organization of the study in all participating centers. JG wrote the study protocol and is responsible for the implementation and organization of the study in all participating centers and the conduct of the database. CD contributed to the design of the study. SV contributed to the design of the study and is responsible for the local conduct of the study in Leuven. PS contributed to the design of the study and is responsible for the local conduct of the study in Bern. MSB contributed to the design of the study and is responsible for the local conduct of the study in San Francisco. MLB contributed to the design of the study and is responsible for the local conduct of the study in The Hague. All authors read and approved the final version of the manuscript.

Ethics approval and consent to participate

The study has been approved Medical Ethics Committee (METC Zuid-West Holland/Erasmus Medical Center; MEC-2020-0812) and is conducted in complicance with the European Union Clinical Trials Directive (2001/20/EC) and the principles of the Declaration of Helsinki (2013).

Consent for publication

By giving written informed consent, patients agree with the storage of data and publication of the study results.

Competing interests

The authors declare that they have no competing interests.

For peer review only

REFERENCES

1. DeAngelis LM (2001) Brain tumors. *N Engl J Med.* 344:114-123
2. Omuro A, DeAngelis LM (2013) Glioblastoma and other malignant gliomas: A clinical review. *JAMA.* 310:1842-1850
3. Stupp R, Mason WP, van den Bent MJ, Weller M, Fisher B, Taphoorn MJ, Belanger K, Brandes AA, Marosi C, Bogdahn U, Curschmann J, Janzer RC, Ludwin SK, Gorlia T, Allgeier A, Lacombe D, Cairncross JG, Eisenhauer E, Mirimanoff RO (2005) Radiotherapy plus concomitant and adjuvant temozolomide for glioblastoma. *N Engl J Med.* 352:987-996
4. Ho VK, Reijneveld JC, Enting RH, Bienfait HP, Baumert BG, Visser O (2014) Changing incidence and improved survival of gliomas. *Eur J Cancer.* 50:2309-2318
5. Eckel-Passow J.E. et al. Glioma groups bsed on 1p/19q, IDH, and TERT promotor mutations in tumors. *N Engl J Med.* 2015. 372:2499-508
6. Taal W, Oosterkamp HM, Walenkamp AM et al. Single-agent bevacizumab or lomustine versus a combination of bevacizumab plus lomustine in patients with recurrent glioblastoma (BELOB trial): a randomized controlled phase 2 trial. *Lancet Oncol.* 2014.15:943-53
7. Stummer W, Pichlmeier U, Meinel T et al. Fluorescence-guided surgery with 5-aminolevulinic acid for resection of malignant glioma: randomized controlled multicentre phase III trial. *Lancet Oncol.* 2006. 7:392-401
8. Smith JS, Cha S, Chang EF, Chang SM, Lamborn KR, Prados MD, Cha S, Tihan T, Vandenberg S, McDermott MW, Berger MS (2008) Role of extent of resection in the long-term outcome of low-grade hemispheric gliomas. *J Clin Oncol.* 26:1338-1345
9. Stummer W, Meinel T, Pichlmeier U, Reulen HJ, Schumacher W, Tonn JC, Rohde V, Oppel F, Turowski B, Woiciechoswky C, Franz K, Pietsch T (2008) Extent of resection and survival in glioblastoma multiforme: Identification of and adjustment for bias. *Neurosurgery.* 62:564-576
10. Sanai N, Berger MS (2008) Glioma extent of resection and its impact on patient outcome. *Neurosurgery*. 62:753-764
11. Vourinen V, Hinkka S, Färkkilä M, Jääskeläinen J, (2003) Debulking or biopsy of malignant glioma in elderly people – randomized study. *Acta Neurochir.* 145:5-10.
12. Lacroix M, Abi-Said S, Fourney DR, Gokaslan ZL, Shi W, DeMonte F, Lang FF, McCutcheon IE, Hassenbusch SJ, Holland E, Hess K, Michael C, Miller D, Sawaya R

For peer review only

(2001) A multivariate analysis of 416 patients with glioblastoma multiforme: prognosis, extent of resection, and survival. *J Neurosurg.* 95:190-198

13. Jeremic B, Grujicic D, Antunovic V, Djuric L, Stojanovic M, Shibamoto Y (1994) Influence of extent of surgery and tumor location on treatment outcome of patients with glioblastoma multiforme treated with combined modality approach. *J Neurooncol.* 21:177-185
14. Keles GE, Anderson B, Berger MS (1999) The effect of extent of resection on time to tumor progression and survival in patients with glioblastoma multiforme of the cerebral hemisphere. *Surg Neurol.* 52:371-379
15. Keles GE, Lamborn KR, Chang SM, Prados MD, Berger MS (2004) Volume of residual disease as a predictor of outcome in adult patients with recurrent supratentorial glioblastomas multiforme who are undergoing chemotherapy. *J Neurosurg.* 100:41-46
16. Van den Bent MJ, Stupp R, Mason W (2005) Impact of the extent of resection on overall survival in newly diagnosed glioblastoma after chemo-irradiation with temozolomide: further analyses of EORTC study 26981. *Eur J Cancer Suppl.* 3:134
17. De Witt Hamer PC, Robles SG, Zwinderman AH, Duffau H, Berger MS (2012) Impact of intraoperative stimulation brain mapping on glioma surgery outcome: a meta-analysis. *J Clin Oncol.* 30:2559-2565
18. Black PM, Ronnen SF (1987). Cortical mapping for defining the limits of tumor resection: *Neurosurgery.* 20:914-919.
19. Gupta DK, Chandra PS, Ohja BK, Sharma BS, Mahapatra AK, Mehta VS (2007) Awake craniotomy versus surgery under general anesthesia for resection of intrinsic lesions of eloquent cortex – a prospective randomized study. *Clin Neurol Neurosurg.* 109:335-343
20. Brown TJ, Brandmeir NJ, Church EW, Brennan MC, Li M, Rakszawski KL, Patel AS, Rizk EB, Suki D, Sawaya R, Glantz M (2016) Association of the extent of resection with survival in glioblastoma: A systematic review and meta-analysis. *JAMA Oncol.* 2:1460-1469
21. Peruzzi P, Bergese SD, Viloria A, Puente EG, Abdel-Rasoul M, Chiocca EA (2011) A retrospective cohort-matched comparison of conscious sedation versus general anesthesia for supratentorial glioma resection. Clinical article. *J Neurosurg.* 114:633-639
22. Sacko O, Lauwers-Cances V, Brauge D, Sesay M, Brenner A, Roux FE (2011) Awake craniotomy vs surgery under general anesthesia for resection of supratentorial

For peer review only

lesions. *Neurosurgery.* 68:1992-1998

23. Hervey-Jumper SL, Li J, Lau D, Molinaro AM, Perry DW, Meng L, Berger MS (2015) Awake craniotomy to maximize glioma resection: methods and technical nuances over a 27-year period. *J Neurosurg.* 123:325-339
24. Danks RA, Aglio LS, Gugino LD, Black PM (2000) Craniotomy under local anesthesia and monitored conscious sedation for the resection of tumors involving eloquent cortex. *J Neurooncol* 49:131-139
25. De Benedictis A, Moritz-Gasser S, Duffau H (2010) Awake mapping optimizes the extent of resection for low-grade gliomas in eloquent areas. *Neurosurgery.* 66:1074-1084
26. Duffau H (2009) A personal consecutive series of surgically treated 51 cases of insular WHO Grade II glioma: advances and limitations. *J Neurosurg.* 110:696-708
27. Duffau H, Capelle L, Denvil D, Sichez N, Gatignol P, Lopes M, Mitchell MC, Sichez JP, Van Effenterre R (2003) Functional recovery after surgical resection of low grade gliomas in eloquent brain: hypothesis of brain compensation. *J Neurol Neurosurg Psychiatry*. 74:901-907
28. Duffau H, Capelle L, Denvil D, Sichez N, Gatignol P, Taillandier L, Lopes M, Mitchell MC, Roche S, Muller JC, Bitar A, Sichez JP, van Effenterre R (2003) Usefulness of intraoperative electrical subcortical mapping during surgery for low-grade gliomas located within eloquent brain regions: functional results in a consecutive series of 103 patients. *J Neurosurg.* 98:764-778
29. Duffau H, Lopes M, Arthuis F, Bitar A, Sichez JP, Van Effenterre R, Capelle L (2005) Contribution of intraoperative electrical stimulations in surgery of low grade gliomas: a comparative study between two series without (1985-96) and with (1996-2003) functional mapping in the same institution. *J Neurol Neurosurg Psychiatry.* 76:845-851
30. Yoshikawa K, Kajiwara K, Morioka J, Fuji M, Tanaka N, Fujisawa H, Kato S, Nomura And S, Suzuki M (2006) Improvement of functional outcome after radical surgery in glioblastoma patients: the efficacy of navigation-guided fence-post procedure and neurophysiological monitoring. *J Neurooncol.* 78:91-97
31. Gerritsen JK, Arends LR, Dirven CM, Schouten JW, Klimek M, Vincent AJ (2017) Impact of intraoperative stimulation mapping and awake craniotomy on high-grade glioma surgery outcome: A meta-analysis. *Acta Neurochirurgica (Wien).* 161:99-107
32. Gerritsen JK, Viëtor CL, Rizopoulos D, Schouten JW, Klimek M, Dirven CM, Vincent AJ (2017) Awake craniotomy versus craniotomy under general anesthesia for

For peer review only

supratentorial high-grade glioma in eloquent areas: A retrospective controlled-matched study. *Acta Neurochirurgica (Wien).* 161:307-15

33. Krieg SM, Shiban E, Droese D et al. Predictive value and safety of intraoperative neurophysiological monitoring with motor evoked potentials in glioma surgery. *Neurosurgery.* 2012. 70:1060-70
34. Raabe A, Beck J, Schucht P et al. Continuous dynamic mapping of the corticospinal tract during surgery of motor eloquent brain tumors: evaluation of a new method. *J Neurosurg.* 2014. 120:1015-24
35. Schucht P, Seidel K, Jilch A et al. A review of monopolar motor mapping and a comprehensive guide to continuous dynamic motor mapping for resection of motor eloquent brain tumors. *Neurochirurgie.* 2017. 63:175-80
36. Costabile JD, Alaswad E, D'Souza S et al. Current applications of diffusion tensor imaging and tractography in intracranial tumor resection. *Front Oncol.* 2019. 9:426. Doi: 10.3389/fonc.2019.00426
37. Caverzasi E, Hervey-Jumper SL, Jordan KM et al. Identifying preoperative language tracts and predicting postoperative functional recovery using HARDI q-ball fiber tractography in patients with gliomas. *J Neurosurg.* 2016. 125:33-45
38. Sawaya R, Hammoud M, Schoppa D et al. Neurosurgical outcomes in a modern series of 400 craniotomies for treatment of parenchymal tumors. *Neurosurgery.* 1998. 42:1044-55

For peer review only

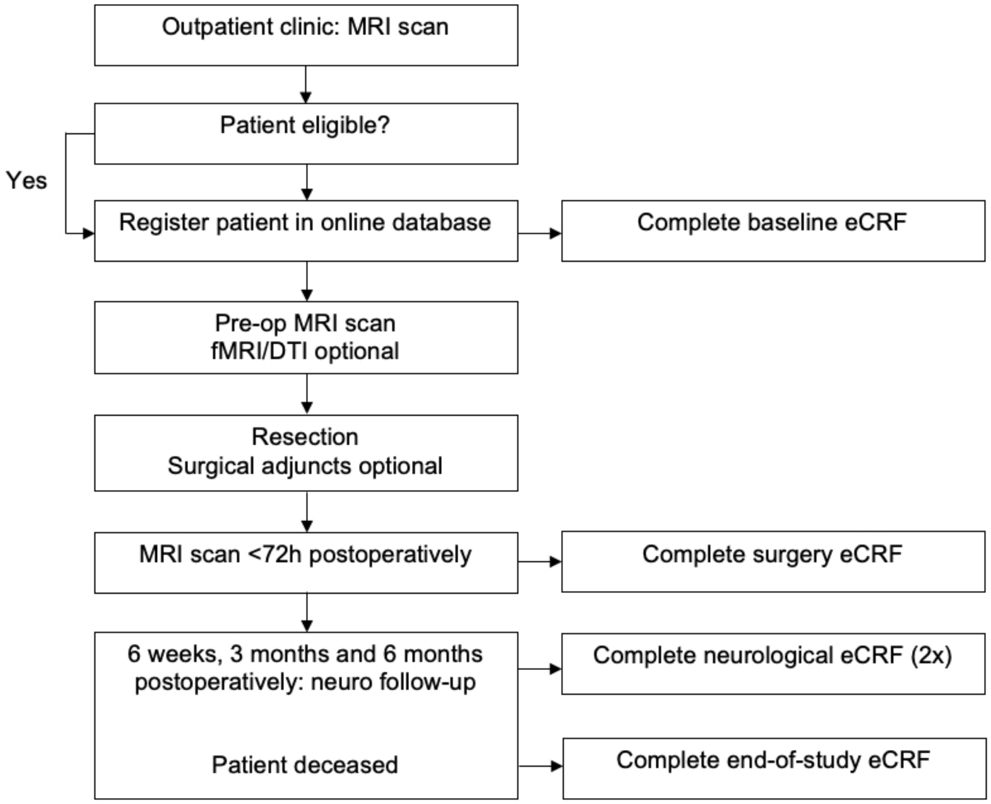

Study Flowchart

279x229mm (72 x 72 DPI)

STROBE Statement—Checklist of items that should be included in reports of ***cohort studies***

	Item No	Recommendation	Page No
**Title and abstract**	1	(*a*) Indicate the study's design with a commonly used term in the title or the abstract	1,2
		(*b*) Provide in the abstract an informative and balanced summary of what was done and what was found	1,2
**Introduction**			
Background/rationale	2	Explain the scientific background and rationale for the investigation being reported	3,4
Objectives	3	State specific objectives, including any prespecified hypotheses	3,4
**Methods**			
Study design	4	Present key elements of study design early in the paper	5
Setting	5	Describe the setting, locations, and relevant dates, including periods of recruitment, exposure, follow-up, and data collection	5
Participants	6	(*a*) Give the eligibility criteria, and the sources and methods of selection of participants. Describe methods of follow-up	5,6
		(*b*) For matched studies, give matching criteria and number of exposed and unexposed	
Variables	7	Clearly define all outcomes, exposures, predictors, potential confounders, and effect modifiers. Give diagnostic criteria, if applicable	5
Data sources/ measurement	8*	For each variable of interest, give sources of data and details of methods of assessment (measurement). Describe comparability of assessment methods if there is more than one group	5,10,11
Bias	9	Describe any efforts to address potential sources of bias	12
Study size	10	Explain how the study size was arrived at	12,13
Quantitative variables	11	Explain how quantitative variables were handled in the analyses. If applicable, describe which groupings were chosen and why	11-13
Statistical methods	12	(*a*) Describe all statistical methods, including those used to control for confounding	13,14
		(*b*) Describe any methods used to examine subgroups and interactions	
		(*c*) Explain how missing data were addressed	
		(*d*) If applicable, explain how loss to follow-up was addressed	
		(*e*) Describe any sensitivity analyses	
**Results**			
Participants	13*	(a) Report numbers of individuals at each stage of study—eg numbers potentially eligible, examined for eligibility, confirmed eligible, included in the study, completing follow-up, and analysed	NA
		(b) Give reasons for non-participation at each stage	
		(c) Consider use of a flow diagram	
Descriptive data	14*	(a) Give characteristics of study participants (eg demographic, clinical, social) and information on exposures and potential confounders	NA
		(b) Indicate number of participants with missing data for each variable of interest	
		(c) Summarise follow-up time (eg, average and total amount)	
Outcome data	15*	Report numbers of outcome events or summary measures over time	NA

For peer review only

Main results	16	(*a*) Give unadjusted estimates and, if applicable, confounder-adjusted estimates and their precision (eg, 95% confidence interval). Make clear which confounders were adjusted for and why they were included<br>(*b*) Report category boundaries when continuous variables were categorized<br>(*c*) If relevant, consider translating estimates of relative risk into absolute risk for a meaningful time period	NA
Other analyses	17	Report other analyses done—eg analyses of subgroups and interactions, and sensitivity analyses	NA
**Discussion**			
Key results	18	Summarise key results with reference to study objectives	NA
Limitations	19	Discuss limitations of the study, taking into account sources of potential bias or imprecision. Discuss both direction and magnitude of any potential bias	16,17
Interpretation	20	Give a cautious overall interpretation of results considering objectives, limitations, multiplicity of analyses, results from similar studies, and other relevant evidence	16,17
Generalisability	21	Discuss the generalisability (external validity) of the study results	16,17
**Other information**			
Funding	22	Give the source of funding and the role of the funders for the present study and, if applicable, for the original study on which the present article is based	1

*Give information separately for exposed and unexposed groups.

Note: An Explanation and Elaboration article discusses each checklist item and gives methodological background and published examples of transparent reporting. The STROBE checklist is best used in conjunction with this article (freely available on the Web sites of PLoS Medicine at http://www.plosmedicine.org/, Annals of Internal Medicine at http://www.annals.org/, and Epidemiology at http://www.epidem.com/). Information on the STROBE Initiative is available at http://www.strobe-statement.org.

For peer review only

BMJ Open

The PROGRAM-study: Awake Mapping versus Asleep Mapping versus No Mapping for High-Grade Glioma Resections: Study Protocol for An International Multicenter Prospective 3-Arm Cohort Study

Journal:	*BMJ Open*
Manuscript ID	bmjopen-2020-047306.R1
Article Type:	Protocol
Date Submitted by the Author:	07-Apr-2021
Complete List of Authors:	Gerritsen, Jasper; Erasmus MC, Department of Neurosurgery<br>Dirven, Clemens; Erasmus MC, Department of Neurosurgery<br>De Vleeschouwer, Steven; Katholieke Universiteit Leuven UZ Leuven, Department of Neurosurgery<br>Jungk, Christine; UniversitatsKlinikum Heidelberg, Neuro-oncology<br>Schucht, Philippe; Inselspital Universitätsspital Bern, Department of Neurosurgery<br>Krieg, Sandro; Technical University of Munich, Department of Neurosurgery<br>Nahed, Brian; Massachusetts General Hospital, Department of Neurosurgery<br>Berger, Mitchel; University of California San Francisco, Department of Neurosurgery<br>Broekman, Marike; Medisch Centrum Haaglanden, Department of Neurosurgery<br>Vincent, Arnaud; Erasmus MC, Department of Neurosurgery
**Primary Subject Heading**:	Neurology
Secondary Subject Heading:	Oncology, Surgery
Keywords:	NEUROSURGERY, SURGERY, Neurological oncology < NEUROLOGY, Neurosurgery < SURGERY

SCHOLARONE™
Manuscripts

I, the Submitting Author has the right to grant and does grant on behalf of all authors of the Work (as defined in the below author licence), an exclusive licence and/or a non-exclusive licence for contributions from authors who are: i) UK Crown employees; ii) where BMJ has agreed a CC-BY licence shall apply, and/or iii) in accordance with the terms applicable for US Federal Government officers or employees acting as part of their official duties; on a worldwide, perpetual, irrevocable, royalty-free basis to BMJ Publishing Group Ltd ("BMJ") its licensees and where the relevant Journal is co-owned by BMJ to the co-owners of the Journal, to publish the Work in this journal and any other BMJ products and to exploit all rights, as set out in our licence.

The Submitting Author accepts and understands that any supply made under these terms is made by BMJ to the Submitting Author unless you are acting as an employee on behalf of your employer or a postgraduate student of an affiliated institution which is paying any applicable article publishing charge ("APC") for Open Access articles. Where the Submitting Author wishes to make the Work available on an Open Access basis (and intends to pay the relevant APC), the terms of reuse of such Open Access shall be governed by a Creative Commons licence – details of these licences and which Creative Commons licence will apply to this Work are set out in our licence referred to above.

Other than as permitted in any relevant BMJ Author's Self Archiving Policies, I confirm this Work has not been accepted for publication elsewhere, is not being considered for publication elsewhere and does not duplicate material already published. I confirm all authors consent to publication of this Work and authorise the granting of this licence.

For peer review only

The PROGRAM-study: Awake Mapping versus Asleep Mapping versus No Mapping for High-Grade Glioma Resections: Study Protocol for An International Multicenter Prospective 3-Arm Cohort Study.

Running head: The PROGRAM-study

Drs. Jasper K.W. Gerritsen[1] MD (Corresponding author)
Prof. dr. Clemens M.F. Dirven[1] MD PhD
Prof. dr. Steven de Vleeschouwer[2] MD PhD
Prof. dr. Philippe Schucht[3] MD PhD
Dr. Christine Jungk[4] MD PhD
Prof. dr. Sandro Krieg[5] MD MBA IFAANS
Dr. Brian V. Nahed[6] MD MSc FACS FAANS
Dr. Mitchel S. Berger[7] MD FACS FAANS
Dr. Marike L.D. Broekman[8] MD PhD
Dr. Arnaud J.P.E. Vincent[1] MD PhD

[1]Department of Neurosurgery, Erasmus Medical Center, Rotterdam, The Netherlands
[2]Department of Neurosurgery, University Hospital Leuven, Belgium
[3]Department of Neurosurgery, Inselspital Universitätsspital Bern, Switzerland
[4]Department of Neurosurgery, University Hospital Heidelberg, Germany
[5]Department of Neurosurgery, Technical University Munich, Germany
[6]Department of Neurosurgery, Massachusetts General Hospital, Boston MA, USA
[7]Department of Neurosurgery, UCSF: University of California, San Francisco CA, USA
[8]Department of Neurosurgery, Haaglanden Medisch Centrum, The Hague, The Netherlands,

Corresponding author
Name: Jasper K.W. Gerritsen MD
Address: Dr. Molewaterplein 40, 3015 GD Rotterdam, The Netherlands
Email: j.gerritsen@erasmusmc.nl

Keywords: Glioblastoma – Neurological morbidity – Extent of resection – Quality of life – Survival
Word count: 5016 - Funding: none
NTR: NL8998 - Clinicaltrials.gov: NCT04708171
Ethics Committee (IRB): Issuing body: Erasmus MC, ID: MEC-2020-0812
Version 3.0 14-09-2020

For peer review only

A B S T R A C T

Introduction

The main surgical dilemma during glioma resections is the surgeon's inability to accurately identify eloquent areas when the patient is under general anesthesia (GA) without mapping techniques. Intraoperative stimulation mapping (ISM) techniques can be used to maximize extent of resection in eloquent areas yet simultaneously minimze the risk of postoperative neurological deficits. ISM has been widely implemented for low-grade glioma resections (LGG) backed with ample scientific evidence, but this is not yet the case for high-grade glioma (HGG) resections. Therefore, ISM could thus be of important value in HGG surgery to improve both surgical and clinical outcomes.

Methods and Analysis

This study is an international, multicenter, prospective 3-arm cohort study of observational nature. Consecutive HGG patients will be operated with awake mapping, asleep mapping or no mapping with a 1:1:1 ratio. Primary endpoints are: 1) proportion of patients with NIHSS (National Institute of Health Stroke Scale) deterioration at 6 weeks, 3 months and 6 months after surgery and 2) residual tumor volume of the contrast-enhancing and non-contrast-enhancing part as assessed by a neuroradiologist on postoperative contrast MRI scans. Secondary endpoints are: 1) overall survival (OS) and 2) progression-free survival (PFS) at 12 months after surgery; 3) onco-functional outcome and 4) frequency and severity of Serious Adverse Events (SAEs) in each arm. Total duration of the study is 5 years. Patient inclusion is 4 years, follow-up is 1 year.

Ethics and Dissemination

The study has been approved by the Medical Ethics Committee (METC Zuid-West Holland/Erasmus Medical Center; MEC-2020-0812). The results will be published in peer-reviewed academic journals and disseminated to patient organisations and media.

Strenghts and limitations

- First multicenter prospective study directly comparing awake mapping, asleep mapping and no mapping for glioblastoma resections in or near eloquent areas.
- International, multicenter design on a large scale, which will be of substantial benefit with regard to subgroup analyses and external generalizability of the results.
- Observational design will not exclude all possible, inherent forms of bias.

For peer review only

INTRODUCTION

Gliomas are the most common malignant tumors of the central nervous system (CNS) and are classified into grades 1-4, where grade 1 and -2 consist of low-grade gliomas (LGG) and grades 3 and -4 represent high-grade gliomas (HGG)[1,2]. Gliomas are relatively rare (incidence of 5/100,000 persons/year in Europe and North America), but are associated with a relatively high morbidity and mortality regardless of years of scientific efforts to improve clinical outcomes in these patients[1-7].

Studies show that maximizing the extent of resection of the contrast-enhancing part – and recently, the non-contrast-enhancing part as well – results in improved patient survival rates[8-15]. Moreover, patients with gross-total resections (GTR) derived the most benefit from the adjuvant chemoradiotherapy compared to patient with subtotal resections[16]. However, in excess of 50% of gliomas are located in- or near eloquent areas of the brain[2]. Eloquent areas are important areas within the brain where speech and/or motor functions are located. Damaging these areas during surgery can lead to severe and permanent neurological deficits that seriously impact the quality of life. As a consequence of this worsened condition, some patients are excluded for radio- and chemotherapy, leading to suboptimal clinical outcomes[16].

Thus, the main surgical problem for the surgeon is the inability to accurately identify these eloquent areas when the patient is under general anesthesia (GA) when no brain mapping techniques are being used. Surgeons often choose a more defensive approach for tumors that are located in or near these areas to prevent postoperative neurological deficits in patients with an already poor prognosis[2,10,12-15]. The use of intraoperative stimulation (neurophysiological) mapping techniques (ISM) can be necessary to enable the surgeon to resect as much tumor as possible while preserving quality of life and neurological functioning in these patients[17]. Mapping of motor-eloquent tumors can be performed while the patient is awake or asleep, while speech mapping can only be performed when the patient is awake. The use of mapping techniques has tremendous potential in glioma resections in eloquent areas, especially for HGG patients. However, there is currently no international consensus regarding the use of these techniques. The scientific evidence for the use of these techniques in this patient group is currently both inconclusive and fragmented. We therefore propose an international, multicenter prospective cohort study in which the use of awake and asleep mapping techniques in HGG patients will be evaluated.

The described research initiative will be able to study these techniques in a prospective setting while covering a breadth of centers and countries. Hence, the data generated in this ENCRAM research collaboration will be able to answer multiple research questions with excellent generalizability, external validity and overall quality in both a cost-effective and practical setting[18].

For peer review only - http://bmjopen.bmj.com/site/about/guidelines.xhtml

METHODS/DESIGN

Study design

This is a international, multicenter, prospective, 3-arm cohort study. Eligible patients are operated using awake mapping, asleep mapping or no mapping with a 1:1:1 ratio with a sequential computer-generated random number as subject ID. Patients with motor-eloquent tumors will be treated in all study arms, while speech-eloquent tumors will only be treated in either the awake mapping or no mapping arm. The PROGRAM study is similar to the SAFE-trial (awake craniotomy versus craniotomy under general anesthesia for glioblastoma patients, NCT03861299) and is initiated by the same center, however, the presented study will be different in various ways: the PROGRAM study (1) will be an observational, prospective cohort study, (2) will include asleep mapping as an additional treatment arm, (3) will evaluate the extent of resection of the non-contrast-enhancing part of the tumor as well, (4) will include both WHO grade III and grade IV gliomas, (5) will include an onco-functional score as one of the outcomes, and (6) will include neurosurgical centers in the United States and is part of the ENCRAM Research Consortium[18].

Study objectives

The primary study objective is to evaluate the safety and efficacy of resections with or without mapping techniques (neurological morbidity and extent of resection) in HGG patients as expressed by NIHSS scores and volumetric data. Secondary study objectives are to study the overall survival (OS), progressive-free survival (PFS) and onco-functional outcome after resections with or without mapping techniques as expressed by survival data, progression on MRI scans and combining postoperative EOR/NIHSS outcomes respectively.

Study setting and participants

Patients will be recruited for the study from the neurosurgical or neurological outpatient clinic or through referral from general hospitals of the participating neurosurgical hospitals, located in Europe and the United States. The study is open to additional participating neurosurgical centers.

Patient and public involvement statement

Patients enrolled in the SAFE-trial (awake craniotomy versus craniotomy under general anesthesia for glioblastoma patients, NCT03861299) were consulted for this study to include patient experiences with resections with- and without mapping.

Inclusion criteria

In order to be eligible to participate in this study, a subject must meet all of the following criteria:

1. Age ≥18 years and ≤90 years

For peer review only - http://bmjopen.bmj.com/site/about/guidelines.xhtml

2. Tumor diagnosed as HGG (WHO grade III/IV) on MRI as assessed by the neurosurgeon
3. Tumors situated in or near eloquent areas; motor cortex, sensory cortex, subcortical pyramidal tract, speech areas or visual areas as indicated on MRI (Sawaya Grading II and II)[19]
4. The tumor is suitable for resection (according to neurosurgeon)
5. Written informed consent

Exclusion criteria

A potential subject who meets any of the following criteria will be excluded from participation in this study:

1. Tumors of the cerebellum, brainstem or midline
2. Multifocal contrast enhancing lesions
3. Medical reasons precluding MRI (e.g. pacemaker)
4. Inability to give written informed consent
5. Secondary high-grade glioma due to malignant transformation from low-grade glioma
6. Second primary malignancy within the past 5 years with the exception of adequately treated in situ carcinoma of any organ or basal cell carcinoma of the skin

Interventions

(1) Awake craniotomy with local anesthesia (arm 1: awake mapping).

On the evening before surgery 1.5–2.0 mg lorazepam is administered for anxiolysis and 2x8 mg dexamethason. The patient is sedated with a bolus injection of propofol (0.5–1 mg/kg) and kept sedated with a propofol infusion pump (mean: 4 mg/kg/h) and remifentanil ((0.5-2 µg/kg/min). Supplemental O_2 might be provided through a nasal cannula. Patients typically receive 1-2 g of cefazolin and sometimes up to 1 g/kg of mannitol (all verified with the surgeon). The room is kept warm and patient covered as the goal is to have the core temperature above 36 C° during motor mapping. An arterial line (with standard monitoring for vital signs in addition to BP monitoring), central venous catheter, and urinary catheter are inserted. The patient is awakened and positioned on the table. At this point local anaesthesia for the fixation of the head in the Mayfield clamp and the surgical field is provided with a mixture of 10 mL lidocaine 2% with 10 mL bupivacaine 0.5% plus adrenaline 1:200,000 for the Mayfield clamp and up to 40 mL bupivacaine 0.375% with adrenaline 1:200,000 for the surgical field. After positioning, clamp fixation, and surgical field infiltration, patients are sedated again for the trephination until the dura mater is opened, after local application of some drops of local anaesthetics. Propofol sedation is stopped after opening of the dura, with the patient awakening with as few external stimuli as possible. Cortical stimulation is performed with a

For peer review only

bipolar electrical stimulator. The distance between both poles is 5 mm, and stimulation is performed by placing this bipolar pincet directly on the cortical surface and stimulating with increasing electrical biphasic currents of 2–12 mA (1-2 mA increasing steps, pulse frequency 60 Hz, single pulse phase duration of 100 microsec.) until motor or speech arrest is observed. For motor mapping a 2-second train and for speech mapping a 5-second train is used, respectively. The Boston naming test and repetition of words is done in cooperation with a neuropsychologist/linguist, who will inform the neurosurgeon of any kind of speech arrest or dysarthria. The difference between these is not always clear, but can be distinguished from involuntary muscle contraction affecting speech. When localizing the motor and sensory cortex, the patient is asked to report any unintended movement or sensation in extremities or face. Confirmed functional cortical areas are marked with a number. After completion of cortical mapping, a resection of the tumour is performed as radical as possible using an ultrasonic aspirator (CUSA) and suction tube, while sparing these functional areas. When the tumour margins or white matter is encountered or when on regular neuronavigation the eloquent white matter tracts are thought to be in close proximity, subcortical stimulation (biphasic currents of 8–16 mA, 1-2 mA increasing steps, pulse frequency 60 Hz, single pulse phase duration of 100 microsec., 2-second train) is performed to localize functional tracts. If subcortical tracts are identified, resection is stopped. During the resection of the lesion close to an eloquent area, the patient is involved in a continuous dialogue with the neuropsychologist. That way the neurosurgeon has ‘online’-control of these eloquent areas. In case of beginning disturbances of communication or of motor or sensory sensations the resection is cessated immediately. When, due to stimulation, an epileptic seizure occurs, this is stopped by administering some drops of iced saline on the just stimulated cortical area.. If a seizure continues, an i.v. propofol or diphantoin bolus of 0.5 mg/kg is administrated and repeated until the seizure stops. The mapping procedure is temporarily halted. If the patient is adequate, cooperative and able to carry out tasks after the seizure, the mapping procedure can continue. In the case of refractory seizures, the mapping procedure will be permanently halted and the resection will continue under general anesthesia. After resection of the tumour a final neurological examination is performed. During closure of the surgical field the patient is sedated with propofol again. After wound closure and dressing, sedation is stopped. The awake patient is transferred to the post-anaesthesia care unit (PACU), where the patient is hemodynamically and neurologically monitored for 24 hours.

(2) Asleep mapping under general anesthesia (arm 2: asleep mapping).

An IV is started on ipsilateral hand to the tumor. The patient is premedicated with up to 2 mg of midazolam. None if altered mental status (prevent further increase in ICP). Arterial (ipsilateral to tumor) catheter is inserted after induction of anesthesia. Anesthesia goals are to decrease ICP (if high), to maintain adequate CPP (at least 70 mmHg) to prevent cerebral ischemia from brain retraction, and to allow intraoperative cortical motor mapping. Patients typically receive 1-2 g of

For peer review only

cefazolin, and 4 mg of decadron before skin incision, and sometimes up to 1 g/kg of mannitol (all verified with the surgeon). The room is kept warm and patient covered as the goal is to have the core temperature above 36 C° during motor mapping. Induction with propofol. In case of increased ICP, have patient hyperventilate during preoxygenation and continue hyperventilation with mask as soon as possible after induction of anesthesia. Fentanyl up to 5 µg/kg in divided doses throughout induction, prior to intubation. Adequate neuromuscular blockade (rocuronium) is verified prior to intubation to avoid coughing/straining. Eyes are taped, and at least one additional large bore IV is inserted. Neuromuscular relaxation is let to wear off for motor mapping (do not reverse). Patient position will depend on location of tumor. Anesthesia is mainained with 70% nitrous oxide in oxygen, low dose inhalation agent (less than 0.5 MAC), and a remifentanil (0.2 µg/kg/min) or fentanyl infusion (2 µg/kg/hr). Euvolemia is maintained (Lactated Ringer's). Mild hyperventilation (PaCO2 35 mmHg) is used. Once the bone flap is removed, the surgeon assesses the tightness of the dura. ICP is further decreased if necessary (pCO2, mannitol, propofol, head up etc.). Once the dura is open, the goal is to avoid brain shift so that stereotactic navigation system can be used optimally. During motor mapping, the arm, leg and face are uncovered to observe for movement. Stimulation is performed with the use of evoked potentials and continuous dynamic mapping/direct subcortical stimulation (CDM/DSS) with a monopolar stimulator (INOMED© Medizintechnik GmBH, Germany). During stimulation, TES-MEP registration is performed of the contralateral m. orbicularis oris, m. orbicularis oculi, m. biceps brachii, m. abductor pollicis, m. rectus femoris and m. tibialis anterior; and the ipsilateral m. abductor pollicis. SSEP registration is performed of the contralateral n. tibialis and bilateral n. medianus. The pulse form is negative, with 5 pulses and a pulse width of 500 µs, ISI 4 and current between 5-20 mA. In case of poststimulation continuation of motor activity, the surgeon will try to stop it by applying cold saline on the cortex. Have propofol (10 mg/ml) in line in case of intraoperative seizures (0.5 mg/kg for seizure suppression). May use neuromuscular relaxants after the last motor mapping. Fentanyl infusion is usually stopped at the beginning of closure. Remifentanil infusion is stopped about 10 min before end of surgery. At this point, use of inhalation agent may be replaced with a propofol infusion (50-100 µg/kg/min). pCO2 is normalized to facilitate spontaneous breathing at the end of the operation. Use of inhalation agents (or propofol) is usually stopped about 10-15 min before end of surgery, and nitrous oxide at the end of surgery. Residual neuromuscular blockade is reversed once the Mayfield pins have been removed. At the end of the procedure all anaesthetics are stopped and patient is brought to the Post Anaesthesia Care Unit (PACU/IC). Detubation of the patient is performed as early as possible, if patient fulfils the detubation criteria (> 36 C body temperature, stable hemodynamics, sufficient spontaneous ventilation, adequate response to verbal orders). Postoperative analgesia is provided with paracetamol i.v. or p.o. 1 g up to 4 dd and morphine 7.5 mg s.c. up to 4 dd, if necessary. At the post-anesthesia care unit (PACU) the patient is hemodynamically and neurologically monitored for 24 hours.

For peer review only - http://bmjopen.bmj.com/site/about/guidelines.xhtml

(3) Craniotomy under general anaesthesia without mapping (arm 3: no mapping).

On the evening before surgery 1.5–2.0 mg lorazepam is administered for anxiolysis. 60 min. before anaesthesia induction the patient receives 1g paracetamol p.o. and 7.5-15 mg midazolam p.o. if requested for sedation. En route to the operating room, 0.5-2 mg midazolam i.v. may be given. 1g cefazoline is given iv. for antibiotic prophylaxis before anaesthesia induction. General anaesthesia is induced intravenously with fentanyl 0.25-0.5 mg, propofol 100-200 mg and cis-atracurium 10-20 mg. After induction of anaesthesia, patient is orotracheally intubated and mechanical ventilation is applied. Respiratory rate and tidal volume are adjusted to keep the patient normocapnic.
An arterial line (alternatively: two peripheral i.v.'s), central venous catheter (v. basilica), and urinary catheter are inserted. Anaesthesia is maintained with propofol (up to 10 mg/kg/h) and remifentanil (0.5-2 μg/kg/min). isoflurane (up to 1 MAC) and clonidine (1-2 μg/kg) may be added for maintenance, if necessary (a beta blocker or calcium channel blocker may be used to control BP as an alternative to clonidine). The fluid management is aiming for normovolemia. 0.9% saline solution and balanced crystalloids are used for maintenance, in case of blood loss > 300 ml, HAES 130/0.4 solution will be given. Temperature management is aiming for normothermia, warm-air blankets and warmed infusion lines are used. Arterial blood gas analysis is performed at the beginning of the procedure and repeated, if necessary. Electrolytes are controlled and substituted and hyperglycemia will be treated with insulin, if necessary. The anesthetized patient is positioned on the table. Local infiltration of the scalp is performed with 20 ml lidocaine 1% with adrenaline 1:200.000 to reduce bleeding. The insertion points of the Mayfield clamp are not infiltrated with local anaesthetics. Trephination and tumour resection are performed without any additional neuro-psychological monitoring, guided by standard neuronavigation. At the end of the procedure all anaesthetics are stopped and patient is brought to the post-anesthesia care unit (PACU). Detubation of the patient is performed as early as possible, if patient fulfils the detubation criteria (>36 C body temperature, stable hemodynamics, sufficient spontaneous ventilation, adequate response to verbal orders). Postoperative analgesia is provided with paracetamol i.v. or p.o. 1 g up to 4 dd and morphine 7.5 mg s.c. up to 4 dd, if necessary. At the PACU the patient is hemodynamically and neurologically monitored for 24 hours.

<u>Surgical adjuncts and additional imaging</u>

The use of fMRI, DTI (Diffusion Tensor Imaging), ultrasound or 5-ALA is allowed to be used in all groups on the surgeon's indication.

<u>Participant timeline</u>

The flow diagram illustrates the main study procedures, including follow-up evaluations (Figure 1). In summary, study patients are allocated to either the awake mapping, asleep mapping or no mapping

For peer review only

For peer review only - http://bmjopen.bmj.com/site/about/guidelines.xhtml

group and will undergo evaluation at presentation (baseline) and during the follow-up period at 6 weeks, 3 months, 6 months and 12 months postoperatively. Motor function will be evaluated using the NIHSS (National Institute of Health Stroke Scale) and MRC (Medical Research Council) scales. Language function will be evaluated using a standard neurolinguistic test-battery consisting of the Aphasia Bedside Check (ABC), Shortened Token test, Verbal fluency and Picture description. Cognitive function will be assessed using the Montreal Cognitive Assessment (MoCA). Patient functioning with be assessed with the Karnofsky Performance Scale (KPS) and the ASA (American Society of Anesthesiologists) physical status classification system. Health-related quality of life (HRQoL) will be assessed with the EQ-5D questionnaire. Overall survival and progression-free survival will be assessed at 12 months postoperatively. We expect to complete patient inclusion in 4 years. The estimated duration of the study (including follow-up) will be 5 years.

Study procedures: Clinical evaluations and follow-up

- Pre-op (baseline) CRF
  - Unique subject ID, demographics (centre, year, gender, age), tumor specific factors (tumor volume pre-op, tumor hemisphere and lobe; eloquent areas), patient specific factors: preoperative KPS, ASA score, neurological status (NIHSS), MRC grade arm/leg, neurolinguistic testing, MOCA, EQ-5D.

- Surgery CRF
  - Type of ISM, surgeon's rationale for modality, use of surgical adjuncts, use of additional imaging, radiological factors: resection percentage (both the contrast-enhancing and non-contrast-enhancing part), residual volume and postoperative ischemia.

- Follow-up CRFs
  - 6 weeks postoperatively: histology and molecular markers (WHO grade, MGMT status, IDH-1 status), neurological status (NIHSS), MRC grade arm/leg, status MRC arm/MRC leg/facialis/speech/visual (new, worsened, improved, stable), KPS, MOCA, EQ-5D.
  - 3 months postoperatively: neurological status (NIHSS), MRC grade arm/leg, status MRC arm/MRC leg/facialis/speech/visual (new, worsened, improved, stable), KPS, neurolinguistic testing, MOCA, EQ-5D.
  - 6 months postoperatively: neurological status (NIHSS), adjuvant treatment, MRC grade arm/leg, status MRC arm/MRC leg/facialis/speech/visual (new, worsened, improved, stable), KPS, MOCA, EQ-5D.

For peer review only - http://bmjopen.bmj.com/site/about/guidelines.xhtml

- 12 months postoperatively: progression-free survival, overall survival (end-of-study).

Outcomes

Primary outcome measures

The primary outcomes are 1) proportion of patients with NIHSS (National Institute of Health Stroke Scale) deterioration at 6 weeks, 3 months and 6 months postoperatively; deterioration is defined as an increase of at least one point on the total NIHSS score compared to this score at baseline and 2) residual tumor volume of the contrast-enhancing and non-contrast enhancing part, as assessed by a neuroradiologist on postoperative T1 with contrast MRI scan sequences using manual or semi-automatic volumetric analyses (Brainlab Elements iPlan CMF Segmentation, Brainlab AG, Munich, Germany; or similar software).

Secondary outcome measures

The secondary outcomes are 1) progression-free survival (PFS) at 12 months defined as time from diagnosis to disease progression (occurrence of a new tumor lesions with a volume greater than 0.175 cm^3, or an increase in residual tumor volume of more than 25%) or death, whichever comes first; 2) overall survival (OS) at 12 months defined as time from diagnosis to death from any cause; 3) onco-functional outcome defined as the calculated coordinate of the EOR on the x-axis and the postoperative NIHSS deterioration on the y-axis and 4) frequency and severity of Serious Adverse Events (SAEs) in each arm.

NIHSS

The National Institutes of Health Stroke Scale, or NIH Stroke Scale (NIHSS) is a tool used by healthcare providers to objectively quantify the impairment caused by a stroke, but has been used extensively for outcome in glioma surgery because of the lack of such scale for neuro-oncologic purposes and has been validated. The NIHSS is composed of 11 items, each of which scores a specific ability between a 0 and 4. For each item, a score of 0 typically indicates normal function in that specific ability, while a higher score is indicative of some level of impairment. The individual scores from each item are summed in order to calculate a patient's total NIHSS score. The maximum possible score is 42 and the minimum score 0.

Aphasia Bedside Check (ABC)

ABC is a short screening test to detect aphasic disturbances at language comprehension and language production level at the main linguistic levels. It consists of 14 items in total. The cut-off score for signs of aphasia is ≤12.

Shortened Token Test

For peer review only - http://bmjopen.bmj.com/site/about/guidelines.xhtml

The shortened Token Test is a test for language comprehension and for the severity of a language disorder. The patient is asked to point and to manipulate geometric forms on verbal commands. It consists of 36 items. The cut-off score is 29.5.

Verbal fluency (category and letter)

Category and letter fluency are tests to assess flexibility of verbal semantic and phonological thought processing, semantic memory and concept generation. The patients is asked to produce words of a given category (animals, professions) or beginning with a given letter (D, A, T) within a limited time span.

Picture description

This is a subtest from the CAT-NL to assess semi-spontaneous speech in an oral and written way (5 minutes each condition). Scoring can be done according to the manual or more thoroughly according to the variables mentioned by Vandenborre et al[20].

Montreal Cognitive Assessment (MOCA)

The MOCA is a cognitive screening test to detect mild impairments across several cognitive domains; attention, verbal memory, language, visuo-constructive skills, conceptual thought, calculation and orientation. The total score is 30, the cut-off score is ≤26.

EQ-5D

The EQ-5D is a standardized questionnaire to assess the general health-related quality of life (HRQoL) in five domains: mobility, self-care, usual activity, pain/discomfort and anxiety/depression. It is developed by the EuroQol Group and can also be used to calculate quality-adjusted life years (QALYs) for cost-utility analyses.

Sample size

This study has two primary endpoints. In order to guarantee that the overall type I error rate does not exceed 5%, we apply a weighted Bonferroni correction for multiple testing. The sample size calculations that follow take that into account. For the first primary endpoint, proportion of patients with neurological deterioration at 6 weeks post- surgery, we assume a deterioration rate of 10% in the control group (arm 3: no mapping), and 3% in the experimental groups (arm 1 and 2: awake and asleep mapping). A two-sample test for proportions with continuity correction requires 411 patients (137 per arm) in total in order to detect the above-mentioned difference of 7% with 80% power at a 4% significance level. For the second primary endpoint, proportion of patients without residual contrast-enhancing tumor on postoperative MRI, we assume a success rate of 25% in the control group (arm 3: no mapping), and 50% in the experimental groups (arm 1 and 2: awake and asleep

For peer review only - http://bmjopen.bmj.com/site/about/guidelines.xhtml

For peer review only

mapping). A two-sample test for proportions with continuity correction requires 188 patients (94 per arm) in total in order to detect the above-mentioned difference of 25% with 80% power at a 1% significance level. In order to power the study for both primary endpoints, we should include the larger required number of patients, i.e. 411. A total of 411 eligible and evaluable patients in three arms allow the difference of 25% in proportion of patients without residual tumor to be detected with 88% power. Taking into account possible ineligibility and withdrawal of consent (we estimate this at 10%), a total of 453 patients will be included (151 patients per arm).

Data collection

All patient data is collected in the electronic data software Castor EDC. This software allows built-in logical checks and validations to promote data quality. Data entry and group allocation is performed by the study coordinator or locally by trained physicians and research nurses under supervision of the local investigator.

Data analysis

All analyses will be according the intention to treat principle, restricted to eligible patients. Patients initially registered but considered ineligible afterwards based on the histological analysis on tissue extracted during surgery, will be excluded from all analyses.

Primary study parameters

The primary endpoints will be analyzed using multivariate logistic regression. Subgroup analyses for tumor grade (WHO grade III/IV), preoperative neurological morbidity, preoperative tumor volume, patient's age (in 10-year age brackets) and tumor location/eloquence will be performed.
We will be including a stratification factor in the primary analysis model with each 10 observed events using the order of prognostic value as mentioned in the paragraph above, where the first 10 events will be used to estimate the effect of the arm. This rule will be applied in case less than 40 patients in total develop neurological deterioration. In the so constructed multivariate logistic regression model the treatment arm effect will be tested at 4% significance level. The primary analysis of proportion of patients without residual contrast-enhancing tumour consist of a multivariate logistic regression, where arm effect is corrected for all minimization factors. In this model the group effect will be tested at 1% significance level. Manual or semiautomatic segmentation will be performed on axial T1 MRI contrast enhanced slices to measure preoperative and postoperative tumor volume. A determination of volumes will be calculated blinded for the treatment group.

Secondary study parameters

The Kaplan-Meier method will be used to estimate PFS and OS proportions per treatment group at

For peer review only

appropriate time points, while the Greenwood estimate of the standard error will be used to construct the corresponding 95% CI. Multivariate cox proportional hazards models will be built for PFS and OS where treatment group effect will be corrected for minimization factors age group (≤55 years vs >55years), KPS (80–90 vs >90), and left or right hemisphere. Additionally, competing risk analysis will be used to calculate cumulative incidence of PFS (with competing risks progression/relapse and death without progression/relapse which add up to 100% at every time point). Onco-functional outcome will be evaluated using a scatter or bubble plot with volumetric data on the x-axis and neurological status (NIHSS) or patient performance (KPS) on the y-axis. SAEs in both groups will be described.

Study monitoring

No scheduled on-site monitoring visits will be performed. Local investigators will remain responsible for the fact that the rights and well-being of patients are protected, the reported trial data are accurate, complete, and verifiable from source documents and the conduct of the trial is in compliance with the currently approved protocol/amendment(s), with GCP, and with the applicable regulatory requirement(s). Direct access to source documentation (medical records) must be allowed for the purpose of verifying that the data recorded in the CRF are consistent with the original source data. No Data Safety Monitoring Board will be installed: all interventions are care-as-usual and patients are allocated without randomisation.

Adverse events (AEs) and serious adverse events (SAEs)

Adverse events are defined as any undesirable experience occurring to a subject during the study, whether or not considered related to neurosurgery. All adverse events reported spontaneously by the subject or observed by the investigator or his staff will be recorded from start of surgery until 6 weeks after surgery. Serious adverse events are any untoward medical occurrence or effect that results in death; is life-threatening (at the time of the event); requires hospitalization or prolongation of existing inpatients' hospitalization; results in persistent or significant disability or incapacity or any other important medical event that did not result in any of the outcomes listed above due to medical or surgical intervention, but could have been based upon appropriate judgement by the investigator. An elective hospital admission will not be considered as a serious adverse event. Most of the (serious) adverse effects of treatments be mainly related to the surgery: post operative pain, nausea and anaemia (in case of massive blood loss), Infections, intracranial haemorrhage, epilepsy, aphasia, paresis/paralysis in arms or/and legs.

Most of the (serious) adverse effects of treatments (awake surgery or surgery under generalised anaesthesia) will be mainly related to the surgery: postoperative pain, nausea and anaemia (in case of massive blood loss), infections, intracranial haemorrhage, epilepsy, aphasia, paresis/paralysis in arms or/and legs. The neurological morbidity is under investigation in this trial and well-known risk /

For peer review only - http://bmjopen.bmj.com/site/about/guidelines.xhtml

complications of the craniotomy and can be attributed to the nature of the operation. Neurosurgical clinics are well adapted to prevent and treat such events. SAEs will be collected through routine data management.

Publication of results

Trial results will be published in an international journal, communicated to neurological and neurosurgical associations and presented at (inter)national congresses.

For peer review only

DISCUSSION

Neurosurgeons face a major dilemma during glioma surgery: maximizing extent of resection while minimizing risk of postoperative neurological deficits. The use of awake or asleep mapping techniques has the potential to equip the surgeon intraoperatively with the needed information to balance these two surgical goals.

A substantial amount of evidence is available on the usefulness of awake mapping to increase resection percentage while preserving quality of life in low-grade glioma patients[21-32]. In contrast, only very few studies have reported the use of awake mapping in high-grade glioma patients, although this technique could be of important value in these patients as well[17,21, 23-25,32]. Recent retrospective evidence showed that glioblastoma patients operated with awake mapping had significant less postoperative neurological morbidity and significantly higher percentage of total resections[33,34]. In patients with motor-eloquent tumors, the use of asleep mapping techniques with evoked potentials or continuous dynamic mapping can be a viable alternative to preserve these functional tracts[35-37].

There is a clear need for solid prospective evidence of the use of these techniques in HGG patients. The presented international neurosurgical research consortium will provide the needed infrastructure to perform ongoing large-scale data collection[18]. This study aims to evaluate whether the use of awake or asleep mapping is the appropriate answer to the surgeon's surgical dilemma during high-grade glioma resections. Furthermore, it will be the first to directly compare awake and asleep mapping techniques in their ability to improve patient outcomes for neurological morbidity, quality of life and survival. Last, using various multivariate analyses, there will be an additional focus on identifying the best surgical choice in subgroups of high-grade glioma patients.

<u>Trial status</u>

The study will start at April 1st, 2021 and is open to additional participating neurosurgical centers.

For peer review only - http://bmjopen.bmj.com/site/about/guidelines.xhtml

Figure 1: Study flow diagram

ABBREVIATIONS

5-ALA: 5-Aminolevulinic Acid

AC: Awake Craniotomy

AE: Adverse Event

CI: Confidence Interval

CTC: Clinical Trial Center

DTI: Diffusion Tensor Imaging

EMC: Erasmus Medical Center

EOR: Extent of Resection

fMRI: Functional Magnetic Resonance Imaging

GA: General Anesthesia

GCP: Good Clinical Practice

GTR: Gross-Total Resection

HMC: Haaglanden Medical Center

HRQoL: Health-related Quality of Life

ISM: Intraoperative Stimulation Mapping

IRB: Institutional Review Board

KPS: Karnofsky Performance Score

LGG: Low-grade glioma

METC: Medical Ethics Committee

MOCA: Montreal Cognitive Assessment

NIHSS: National Institute of Health Stroke Scale

OS: Overall survival

PACU: Post-Anesthesia Care Unit

PFS: Progression-free survival

SAE: Serious Adverse Event

STR: Subtotal Resection

UCSF: Univeristy of California, San Francisco

WHO: World Health Organization

For peer review only

DECLARATIONS

Author contributions

JG, AV and MLB designed the study, wrote the study protocol and are end-responsible for the implementation and organization of the study in all participating centers. JG wrote the study protocol and is responsible for the implementation and organization of the study in all participating centers and the conduct of the database. CD contributed to the design of the study. SV contributed to the design of the study and is responsible for the local conduct of the study in Leuven. PS contributed to the design of the study and is responsible for the local conduct of the study in Bern. CJ contributed to the design of the study and is responsible for the local conduct of the study in Heidelberg. SK contributed to the design of the study and is responsible for the local conduct of the study in Munich. BN contributed to the design of the study and is responsible for the local conduct of the study in Boston. MSB contributed to the design of the study and is responsible for the local conduct of the study in San Francisco. MLB contributed to the design of the study and is responsible for the local conduct of the study in The Hague. All authors read and approved the final version of the manuscript.

Ethics approval and consent to participate

The study has been approved Medical Ethics Committee (IRB/METC Zuid-West Holland/Erasmus Medical Center; MEC-2020-0812) and is conducted in complicance with the European Union Clinical Trials Directive (2001/20/EC) and the principles of the Declaration of Helsinki (2013).

Consent for publication

By giving written informed consent, patients agree with the storage of data and publication of the study results.

Competing interests

The authors declare that they have no competing interests.

For peer review only

REFERENCES

1. DeAngelis LM (2001) Brain tumors. *N Engl J Med.* 344:114-123
2. Omuro A, DeAngelis LM (2013) Glioblastoma and other malignant gliomas: A clinical review. *JAMA.* 310:1842-1850
3. Stupp R, Mason WP, van den Bent MJ, Weller M, Fisher B, Taphoorn MJ, Belanger K, Brandes AA, Marosi C, Bogdahn U, Curschmann J, Janzer RC, Ludwin SK, Gorlia T, Allgeier A, Lacombe D, Cairncross JG, Eisenhauer E, Mirimanoff RO (2005) Radiotherapy plus concomitant and adjuvant temozolomide for glioblastoma. *N Engl J Med.* 352:987-996
4. Ho VK, Reijneveld JC, Enting RH, Bienfait HP, Baumert BG, Visser O (2014) Changing incidence and improved survival of gliomas. *Eur J Cancer.* 50:2309-2318
5. Eckel-Passow J.E. et al. Glioma groups based on 1p/19q, IDH, and TERT promotor mutations in tumors. *N Engl J Med.* 2015. 372:2499-508
6. Taal W, Oosterkamp HM, Walenkamp AM et al. Single-agent bevacizumab or lomustine versus a combination of bevacizumab plus lomustine in patients with recurrent glioblastoma (BELOB trial): a randomized controlled phase 2 trial. *Lancet Oncol.* 2014.15:943-53
7. Stummer W, Pichlmeier U, Meinel T et al. Fluorescence-guided surgery with 5-aminolevulinic acid for resection of malignant glioma: randomized controlled multicentre phase III trial. *Lancet Oncol.* 2006. 7:392-401
8. Smith JS, Cha S, Chang EF, Chang SM, Lamborn KR, Prados MD, Cha S, Tihan T, Vandenberg S, McDermott MW, Berger MS (2008) Role of extent of resection in the long-term outcome of low-grade hemispheric gliomas. *J Clin Oncol.* 26:1338-1345
9. Stummer W, Meinel T, Pichlmeier U, Reulen HJ, Schumacher W, Tonn JC, Rohde V, Oppel F, Turowski B, Woiciechoswky C, Franz K, Pietsch T (2008) Extent of resection and survival in glioblastoma multiforme: Identification of and adjustment for bias. *Neurosurgery.* 62:564-576
10. Sanai N, Berger MS (2008) Glioma extent of resection and its impact on patient outcome. *Neurosurgery*. 62:753-764
11. Vourinen V, Hinkka S, Färkkilä M, Jääskeläinen J, (2003) Debulking or biopsy of malignant glioma in elderly people – randomized study. *Acta Neurochir.* 145:5-10.
12. Lacroix M, Abi-Said S, Fourney DR, Gokaslan ZL, Shi W, DeMonte F, Lang FF, McCutcheon IE, Hassenbusch SJ, Holland E, Hess K, Michael C, Miller D, Sawaya R (2001) A multivariate analysis of 416 patients with glioblastoma multiforme: prognosis, extent of resection, and survival. *J Neurosurg.* 95:190-198
13. Molinaro AM, Hervey-Jumper S, Morshed RA et al (2020) Association of maximal extent of resection of contrast-enhanced and non-contrast-enhanced tumor with survival within molecular subgroups of patients with newly diagnosed glioblastoma. *JAMA Oncol.* 6:495-503.

For peer review only - http://bmjopen.bmj.com/site/about/guidelines.xhtml

14. Keles GE, Anderson B, Berger MS (1999) The effect of extent of resection on time to tumor progression and survival in patients with glioblastoma multiforme of the cerebral hemisphere. *Surg Neurol.* 52:371-379
15. Keles GE, Lamborn KR, Chang SM, Prados MD, Berger MS (2004) Volume of residual disease as a predictor of outcome in adult patients with recurrent supratentorial glioblastomas multiforme who are undergoing chemotherapy. *J Neurosurg.* 100:41-46
16. Van den Bent MJ, Stupp R, Mason W (2005) Impact of the extent of resection on overall survival in newly diagnosed glioblastoma after chemo-irradiation with temozolomide: further analyses of EORTC study 26981. *Eur J Cancer Suppl.* 3:134
17. De Witt Hamer PC, Robles SG, Zwinderman AH, Duffau H, Berger MS (2012) Impact of intraoperative stimulation brain mapping on glioma surgery outcome: a meta-analysis. *J Clin Oncol.* 30:2559-2565
18. Gerritsen JKW, Broekman MLD, de Vleeschouwer S et al (2021) Letter: The European and North American Consortium and Registry for intraoperative stimulation mapping: framework for a transatlantic collaborative research initiative. *Neurosurgery.* 88:E369. doi: 10.1093/neuros/nyaa568
19. Sawaya R, Hammoud M, Schoppa D et al. Neurosurgical outcomes in a modern series of 400 craniotomies for treatment of parenchymal tumors. *Neurosurgery.* 1998. 42:1044-55
20. Vandenborre D, Visch-Brink E, van Dun K et al. Oral and written picture description in individuals with aphasia. *Int J Lang Commun Disord.* 2018. 53:294-307
21. Gupta DK, Chandra PS, Ohja BK, Sharma BS, Mahapatra AK, Mehta VS (2007) Awake craniotomy versus surgery under general anesthesia for resection of intrinsic lesions of eloquent cortex – a prospective randomized study. *Clin Neurol Neurosurg.* 109:335-343
22. Brown TJ, Brandmeir NJ, Church EW, Brennan MC, Li M, Rakszawski KL, Patel AS, Rizk EB, Suki D, Sawaya R, Glantz M (2016) Association of the extent of resection with survival in glioblastoma: A systematic review and meta-analysis. *JAMA Oncol.* 2:1460-1469
23. Peruzzi P, Bergese SD, Viloria A, Puente EG, Abdel-Rasoul M, Chiocca EA (2011) A retrospective cohort-matched comparison of conscious sedation versus general anesthesia for supratentorial glioma resection. Clinical article. *J Neurosurg.* 114:633-639
24. Sacko O, Lauwers-Cances V, Brauge D, Sesay M, Brenner A, Roux FE (2011) Awake craniotomy vs surgery under general anesthesia for resection of supratentorial lesions. *Neurosurgery.* 68:1992-1998
25. Hervey-Jumper SL, Li J, Lau D, Molinaro AM, Perry DW, Meng L, Berger MS (2015) Awake craniotomy to maximize glioma resection: methods and technical nuances over a 27-year period. *J Neurosurg.* 123:325-339

For peer review only

26. Danks RA, Aglio LS, Gugino LD, Black PM (2000) Craniotomy under local anesthesia and monitored conscious sedation for the resection of tumors involving eloquent cortex. *J Neurooncol* 49:131-139

27. De Benedictis A, Moritz-Gasser S, Duffau H (2010) Awake mapping optimizes the extent of resection for low-grade gliomas in eloquent areas. *Neurosurgery.* 66:1074-1084

28. Duffau H (2009) A personal consecutive series of surgically treated 51 cases of insular WHO Grade II glioma: advances and limitations. *J Neurosurg.* 110:696-708

29. Duffau H, Capelle L, Denvil D, Sichez N, Gatignol P, Lopes M, Mitchell MC, Sichez JP, Van Effenterre R (2003) Functional recovery after surgical resection of low grade gliomas in eloquent brain: hypothesis of brain compensation. *J Neurol Neurosurg Psychiatry*. 74:901-907

30. Duffau H, Capelle L, Denvil D, Sichez N, Gatignol P, Taillandier L, Lopes M, Mitchell MC, Roche S, Muller JC, Bitar A, Sichez JP, van Effenterre R (2003) Usefulness of intraoperative electrical subcortical mapping during surgery for low-grade gliomas located within eloquent brain regions: functional results in a consecutive series of 103 patients. *J Neurosurg.* 98:764-778

31. Duffau H, Lopes M, Arthuis F, Bitar A, Sichez JP, Van Effenterre R, Capelle L (2005) Contribution of intraoperative electrical stimulations in surgery of low grade gliomas: a comparative study between two series without (1985-96) and with (1996-2003) functional mapping in the same institution. *J Neurol Neurosurg Psychiatry.* 76:845-851

32. Yoshikawa K, Kajiwara K, Morioka J, Fuji M, Tanaka N, Fujisawa H, Kato S, Nomura And S, Suzuki M (2006) Improvement of functional outcome after radical surgery in glioblastoma patients: the efficacy of navigation-guided fence-post procedure and neurophysiological monitoring. *J Neurooncol.* 78:91-97

33. Gerritsen JK, Arends LR, Dirven CM, Schouten JW, Klimek M, Vincent AJ (2017) Impact of intraoperative stimulation mapping and awake craniotomy on high-grade glioma surgery outcome: A meta-analysis. *Acta Neurochirurgica (Wien).* 161:99-107

34. Gerritsen JK, Viëtor CL, Rizopoulos D, Schouten JW, Klimek M, Dirven CM, Vincent AJ (2017) Awake craniotomy versus craniotomy under general anesthesia for supratentorial high-grade glioma in eloquent areas: A retrospective controlled-matched study. *Acta Neurochirurgica (Wien).* 161:307-15

35. Krieg SM, Shiban E, Droese D et al. Predictive value and safety of intraoperative neurophysiological monitoring with motor evoked potentials in glioma surgery. *Neurosurgery.* 2012. 70:1060-70

36. Raabe A, Beck J, Schucht P et al. Continuous dynamic mapping of the corticospinal tract during surgery of motor eloquent brain tumors: evaluation of a new method. *J Neurosurg.* 2014. 120:1015-24

For peer review only - http://bmjopen.bmj.com/site/about/guidelines.xhtml

37. Schucht P, Seidel K, Jilch A et al. A review of monopolar motor mapping and a comprehensive guide to continuous dynamic motor mapping for resection of motor eloquent brain tumors. *Neurochirurgie.* 2017. 63:175-80

For peer review only

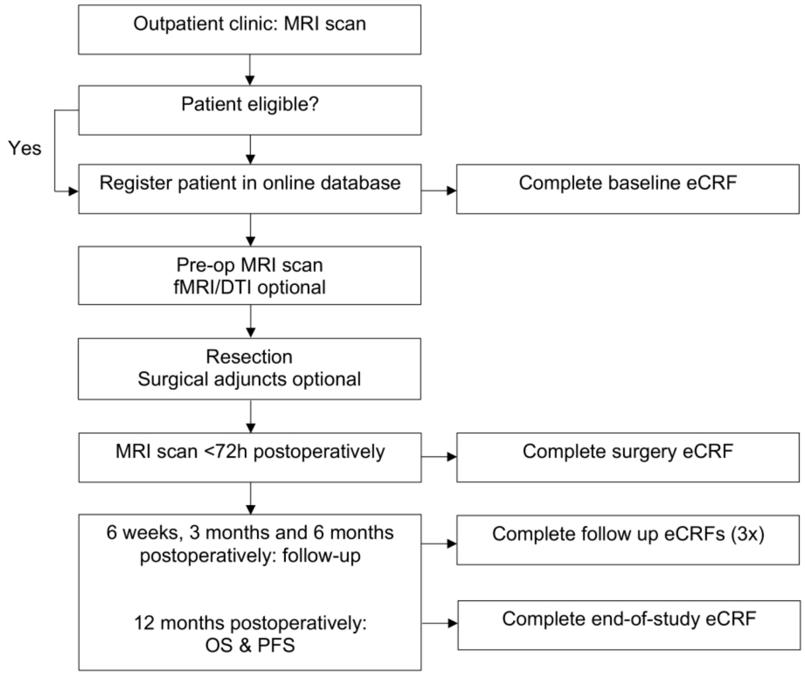

Figure 1: Study flowchart

209x297mm (300 x 300 DPI)

STROBE Statement—Checklist of items that should be included in reports of ***cohort studies***

	Item No	Recommendation	Page No
**Title and abstract**	1	(*a*) Indicate the study's design with a commonly used term in the title or the abstract	1,2
		(*b*) Provide in the abstract an informative and balanced summary of what was done and what was found	1,2
**Introduction**			
Background/rationale	2	Explain the scientific background and rationale for the investigation being reported	3,4
Objectives	3	State specific objectives, including any prespecified hypotheses	3,4
**Methods**			
Study design	4	Present key elements of study design early in the paper	5
Setting	5	Describe the setting, locations, and relevant dates, including periods of recruitment, exposure, follow-up, and data collection	5
Participants	6	(*a*) Give the eligibility criteria, and the sources and methods of selection of participants. Describe methods of follow-up	5,6
		(*b*) For matched studies, give matching criteria and number of exposed and unexposed	
Variables	7	Clearly define all outcomes, exposures, predictors, potential confounders, and effect modifiers. Give diagnostic criteria, if applicable	5
Data sources/ measurement	8*	For each variable of interest, give sources of data and details of methods of assessment (measurement). Describe comparability of assessment methods if there is more than one group	5,10,11
Bias	9	Describe any efforts to address potential sources of bias	12
Study size	10	Explain how the study size was arrived at	12,13
Quantitative variables	11	Explain how quantitative variables were handled in the analyses. If applicable, describe which groupings were chosen and why	11-13
Statistical methods	12	(*a*) Describe all statistical methods, including those used to control for confounding	13,14
		(*b*) Describe any methods used to examine subgroups and interactions	
		(*c*) Explain how missing data were addressed	
		(*d*) If applicable, explain how loss to follow-up was addressed	
		(*e*) Describe any sensitivity analyses	
**Results**			
Participants	13*	(a) Report numbers of individuals at each stage of study—eg numbers potentially eligible, examined for eligibility, confirmed eligible, included in the study, completing follow-up, and analysed	NA
		(b) Give reasons for non-participation at each stage	
		(c) Consider use of a flow diagram	
Descriptive data	14*	(a) Give characteristics of study participants (eg demographic, clinical, social) and information on exposures and potential confounders	NA
		(b) Indicate number of participants with missing data for each variable of interest	
		(c) Summarise follow-up time (eg, average and total amount)	
Outcome data	15*	Report numbers of outcome events or summary measures over time	NA

For peer review only - http://bmjopen.bmj.com/site/about/guidelines.xhtml

Main results	16	(*a*) Give unadjusted estimates and, if applicable, confounder-adjusted estimates and their precision (eg, 95% confidence interval). Make clear which confounders were adjusted for and why they were included<br>(*b*) Report category boundaries when continuous variables were categorized<br>(*c*) If relevant, consider translating estimates of relative risk into absolute risk for a meaningful time period	NA
Other analyses	17	Report other analyses done—eg analyses of subgroups and interactions, and sensitivity analyses	NA
**Discussion**			
Key results	18	Summarise key results with reference to study objectives	NA
Limitations	19	Discuss limitations of the study, taking into account sources of potential bias or imprecision. Discuss both direction and magnitude of any potential bias	16,17
Interpretation	20	Give a cautious overall interpretation of results considering objectives, limitations, multiplicity of analyses, results from similar studies, and other relevant evidence	16,17
Generalisability	21	Discuss the generalisability (external validity) of the study results	16,17
**Other information**			
Funding	22	Give the source of funding and the role of the funders for the present study and, if applicable, for the original study on which the present article is based	1

*Give information separately for exposed and unexposed groups.

Note: An Explanation and Elaboration article discusses each checklist item and gives methodological background and published examples of transparent reporting. The STROBE checklist is best used in conjunction with this article (freely available on the Web sites of PLoS Medicine at http://www.plosmedicine.org/, Annals of Internal Medicine at http://www.annals.org/, and Epidemiology at http://www.epidem.com/). Information on the STROBE Initiative is available at http://www.strobe-statement.org.

For peer review only

For peer review only - http://bmjopen.bmj.com/site/about/guidelines.xhtml

BMJ Open

The PROGRAM-study: Awake Mapping versus Asleep Mapping versus No Mapping for High-Grade Glioma Resections: Study Protocol for An International Multicenter Prospective 3-Arm Cohort Study

Journal:	*BMJ Open*
Manuscript ID	bmjopen-2020-047306.R2
Article Type:	Protocol
Date Submitted by the Author:	29-Jun-2021
Complete List of Authors:	Gerritsen, Jasper; Erasmus MC, Department of Neurosurgery<br>Dirven, Clemens; Erasmus MC, Department of Neurosurgery<br>De Vleeschouwer, Steven; Katholieke Universiteit Leuven UZ Leuven, Department of Neurosurgery<br>Schucht, Philippe; Inselspital Universitätsspital Bern, Department of Neurosurgery<br>Jungk, Christine; UniversitatsKlinikum Heidelberg, Neuro-oncology<br>Krieg, Sandro; Technical University of Munich, Department of Neurosurgery<br>Nahed, Brian; Massachusetts General Hospital, Department of Neurosurgery<br>Berger, Mitchel; University of California San Francisco, Department of Neurosurgery<br>Broekman, Marike; Medisch Centrum Haaglanden, Department of Neurosurgery<br>Vincent, Arnaud; Erasmus MC, Department of Neurosurgery
**Primary Subject Heading**:	Neurology
Secondary Subject Heading:	Oncology, Surgery
Keywords:	NEUROSURGERY, SURGERY, Neurological oncology < NEUROLOGY, Neurosurgery < SURGERY

SCHOLARONE™
Manuscripts

I, the Submitting Author has the right to grant and does grant on behalf of all authors of the Work (as defined in the below author licence), an exclusive licence and/or a non-exclusive licence for contributions from authors who are: i) UK Crown employees; ii) where BMJ has agreed a CC-BY licence shall apply, and/or iii) in accordance with the terms applicable for US Federal Government officers or employees acting as part of their official duties; on a worldwide, perpetual, irrevocable, royalty-free basis to BMJ Publishing Group Ltd ("BMJ") its licensees and where the relevant Journal is co-owned by BMJ to the co-owners of the Journal, to publish the Work in this journal and any other BMJ products and to exploit all rights, as set out in our licence.

The Submitting Author accepts and understands that any supply made under these terms is made by BMJ to the Submitting Author unless you are acting as an employee on behalf of your employer or a postgraduate student of an affiliated institution which is paying any applicable article publishing charge ("APC") for Open Access articles. Where the Submitting Author wishes to make the Work available on an Open Access basis (and intends to pay the relevant APC), the terms of reuse of such Open Access shall be governed by a Creative Commons licence – details of these licences and which Creative Commons licence will apply to this Work are set out in our licence referred to above.

Other than as permitted in any relevant BMJ Author's Self Archiving Policies, I confirm this Work has not been accepted for publication elsewhere, is not being considered for publication elsewhere and does not duplicate material already published. I confirm all authors consent to publication of this Work and authorise the granting of this licence.

For peer review only

The PROGRAM-study: Awake Mapping versus Asleep Mapping versus No Mapping for High-Grade Glioma Resections: Study Protocol for An International Multicenter Prospective 3-Arm Cohort Study.

Running head: The PROGRAM-study

Drs. Jasper K.W. Gerritsen[1] MD (Corresponding author)
Prof. dr. Clemens M.F. Dirven[1] MD PhD
Prof. dr. Steven de Vleeschouwer[2] MD PhD
Prof. dr. Philippe Schucht[3] MD PhD
Dr. Christine Jungk[4] MD PhD
Prof. dr. Sandro Krieg[5] MD MBA IFAANS
Dr. Brian V. Nahed[6] MD MSc FACS FAANS
Dr. Mitchel S. Berger[7] MD FACS FAANS
Dr. Marike L.D. Broekman[8] MD PhD
Dr. Arnaud J.P.E. Vincent[1] MD PhD

[1]Department of Neurosurgery, Erasmus Medical Center, Rotterdam, The Netherlands
[2]Department of Neurosurgery, University Hospital Leuven, Belgium
[3]Department of Neurosurgery, Inselspital Universitätsspital Bern, Switzerland
[4]Department of Neurosurgery, University Hospital Heidelberg, Germany
[5]Department of Neurosurgery, Technical University Munich, Germany
[6]Department of Neurosurgery, Massachusetts General Hospital, Boston MA, USA
[7]Department of Neurosurgery, UCSF: University of California, San Francisco CA, USA
[8]Department of Neurosurgery, Haaglanden Medisch Centrum, The Hague, The Netherlands,

Corresponding author
Name: Jasper K.W. Gerritsen MD
Address: Dr. Molewaterplein 40, 3015 GD Rotterdam, The Netherlands
Email: j.gerritsen@erasmusmc.nl

Keywords: Glioblastoma – Neurological morbidity – Extent of resection – Quality of life – Survival
Word count: 5016 - Funding: none
NTR: NL8998 - Clinicaltrials.gov: NCT04708171
Ethics Committee (IRB): Issuing body: Erasmus MC, ID: MEC-2020-0812
Version 3.0 14-09-2020

For peer review only

ABSTRACT

Introduction

The main surgical dilemma during glioma resections is the surgeon's inability to accurately identify eloquent areas when the patient is under general anesthesia (GA) without mapping techniques. Intraoperative stimulation mapping (ISM) techniques can be used to maximize extent of resection in eloquent areas yet simultaneously minimze the risk of postoperative neurological deficits. ISM has been widely implemented for low-grade glioma resections (LGG) backed with ample scientific evidence, but this is not yet the case for high-grade glioma (HGG) resections. Therefore, ISM could thus be of important value in HGG surgery to improve both surgical and clinical outcomes.

Methods and Analysis

This study is an international, multicenter, prospective 3-arm cohort study of observational nature. Consecutive HGG patients will be operated with awake mapping, asleep mapping or no mapping with a 1:1:1 ratio. Primary endpoints are: 1) proportion of patients with NIHSS (National Institute of Health Stroke Scale) deterioration at 6 weeks, 3 months and 6 months after surgery and 2) residual tumor volume of the contrast-enhancing and non-contrast-enhancing part as assessed by a neuroradiologist on postoperative contrast MRI scans. Secondary endpoints are: 1) overall survival (OS) and 2) progression-free survival (PFS) at 12 months after surgery; 3) onco-functional outcome and 4) frequency and severity of Serious Adverse Events (SAEs) in each arm. Total duration of the study is 5 years. Patient inclusion is 4 years, follow-up is 1 year.

Ethics and Dissemination

The study has been approved by the Medical Ethics Committee (METC Zuid-West Holland/Erasmus Medical Center; MEC-2020-0812). The results will be published in peer-reviewed academic journals and disseminated to patient organisations and media.

Strenghts and limitations

- First multicenter prospective study directly comparing awake mapping, asleep mapping and no mapping for glioblastoma resections in or near eloquent areas.
- International, multicenter design on a large scale, which will be of substantial benefit with regard to subgroup analyses and external generalizability of the results.
- Observational design will not exclude all possible, inherent forms of bias.

For peer review only

INTRODUCTION

Gliomas are the most common malignant tumors of the central nervous system (CNS) and are classified into grades 1-4, where grade 1 and -2 consist of low-grade gliomas (LGG) and grades 3 and -4 represent high-grade gliomas (HGG)[1,2]. Gliomas are relatively rare (incidence of 5/100,000 persons/year in Europe and North America), but are associated with a relatively high morbidity and mortality regardless of years of scientific efforts to improve clinical outcomes in these patients[1-7].

Studies show that maximizing the extent of resection of the contrast-enhancing part – and recently, the non-contrast-enhancing part as well – results in improved patient survival rates[8-15]. Moreover, patients with gross-total resections (GTR) derived the most benefit from the adjuvant chemoradiotherapy compared to patient with subtotal resections[16]. However, in excess of 50% of gliomas are located in- or near eloquent areas of the brain[2]. Eloquent areas are important areas within the brain where speech and/or motor functions are located. Damaging these areas during surgery can lead to severe and permanent neurological deficits that seriously impact the quality of life. As a consequence of this worsened condition, some patients are excluded for radio- and chemotherapy, leading to suboptimal clinical outcomes[16].

Thus, the main surgical problem for the surgeon is the inability to accurately identify these eloquent areas when the patient is under general anesthesia (GA) when no brain mapping techniques are being used. Surgeons often choose a more defensive approach for tumors that are located in or near these areas to prevent postoperative neurological deficits in patients with an already poor prognosis[2,10,12-15]. The use of intraoperative stimulation (neurophysiological) mapping techniques (ISM) can be necessary to enable the surgeon to resect as much tumor as possible while preserving quality of life and neurological functioning in these patients[17]. Mapping of motor-eloquent tumors can be performed while the patient is awake or asleep, while speech mapping can only be performed when the patient is awake. The use of mapping techniques has tremendous potential in glioma resections in eloquent areas, especially for HGG patients. However, there is currently no international consensus regarding the use of these techniques. The scientific evidence for the use of these techniques in this patient group is currently both inconclusive and fragmented. We therefore propose an international, multicenter prospective cohort study in which the use of awake and asleep mapping techniques in HGG patients will be evaluated.

The described research initiative will be able to study these techniques in a prospective setting while covering a breadth of centers and countries. Hence, the data generated in this ENCRAM research collaboration will be able to answer multiple research questions with excellent generalizability, external validity and overall quality in both a cost-effective and practical setting[18].

For peer review only - http://bmjopen.bmj.com/site/about/guidelines.xhtml

METHODS AND ANALYSIS

Study design

This is a international, multicenter, prospective, 3-arm cohort study (registration: clinicaltrials.gov ID number NCT04708171). Eligible patients are operated using awake mapping, asleep mapping or no mapping with a 1:1:1 ratio with a sequential computer-generated random number as subject ID. Patients with motor-eloquent tumors will be treated in all study arms, while speech-eloquent tumors will only be treated in either the awake mapping or no mapping arm. The PROGRAM study is similar to the SAFE-trial (awake craniotomy versus craniotomy under general anesthesia for glioblastoma patients, NCT03861299) and is initiated by the same center, however, the presented study will be different in various ways: the PROGRAM study (1) will be an observational, prospective cohort study, (2) will include asleep mapping as an additional treatment arm, (3) will evaluate the extent of resection of the non-contrast-enhancing part of the tumor as well, (4) will include both WHO grade III and grade IV gliomas, (5) will include an onco-functional score as one of the outcomes, and (6) will include neurosurgical centers in the United States and is part of the ENCRAM Research Consortium[18].

Study objectives

The primary study objective is to evaluate the safety and efficacy of resections with or without mapping techniques (neurological morbidity and extent of resection) in HGG patients as expressed by NIHSS scores and volumetric data. Secondary study objectives are to study the overall survival (OS), progressive-free survival (PFS) and onco-functional outcome after resections with or without mapping techniques as expressed by survival data, progression on MRI scans and combining postoperative EOR/NIHSS outcomes respectively.

Study setting and participants

Patients will be recruited for the study from the neurosurgical or neurological outpatient clinic or through referral from general hospitals of the participating neurosurgical hospitals, located in Europe and the United States. The study is open to additional participating neurosurgical centers.

Patient and public involvement statement

Patients enrolled in the SAFE-trial (awake craniotomy versus craniotomy under general anesthesia for glioblastoma patients, NCT03861299) were consulted for this study to include patient experiences with resections with- and without mapping.

Inclusion criteria

In order to be eligible to participate in this study, a subject must meet all of the following criteria:

For peer review only - http://bmjopen.bmj.com/site/about/guidelines.xhtml

1. Age ≥18 years and ≤90 years
2. Tumor diagnosed as HGG (WHO grade III/IV) on MRI as assessed by the neurosurgeon
3. Tumors situated in or near eloquent areas; motor cortex, sensory cortex, subcortical pyramidal tract, speech areas or visual areas as indicated on MRI (Sawaya Grading II and II)[19]
4. The tumor is suitable for resection (according to neurosurgeon)
5. Written informed consent

<u>Exclusion criteria</u>

A potential subject who meets any of the following criteria will be excluded from participation in this study:

1. Tumors of the cerebellum, brainstem or midline
2. Multifocal contrast enhancing lesions
3. Medical reasons precluding MRI (e.g. pacemaker)
4. Inability to give written informed consent
5. Secondary high-grade glioma due to malignant transformation from low-grade glioma
6. Second primary malignancy within the past 5 years with the exception of adequately treated in situ carcinoma of any organ or basal cell carcinoma of the skin

<u>Interventions</u>

(1) Awake craniotomy with local anesthesia (arm 1: awake mapping).

On the evening before surgery 1.5–2.0 mg lorazepam is administered for anxiolysis and 2x8 mg dexamethason. The patient is sedated with a bolus injection of propofol (0.5–1 mg/kg) and kept sedated with a propofol infusion pump (mean: 4 mg/kg/h) and remifentanil ((0.5-2 μg/kg/min). Supplemental O_2 might be provided through a nasal cannula. Patients typically receive 1-2 g of cefazolin and sometimes up to 1 g/kg of mannitol (all verified with the surgeon). The room is kept warm and patient covered as the goal is to have the core temperature above 36 C° during motor mapping. An arterial line (with standard monitoring for vital signs in addition to BP monitoring), central venous catheter, and urinary catheter are inserted. The patient is awakened and positioned on the table. At this point local anaesthesia for the fixation of the head in the Mayfield clamp and the surgical field is provided with a mixture of 10 mL lidocaine 2% with 10 mL bupivacaine 0.5% plus adrenaline 1:200,000 for the Mayfield clamp and up to 40 mL bupivacaine 0.375% with adrenaline 1:200,000 for the surgical field. After positioning, clamp fixation, and surgical field infiltration, patients are sedated again for the trephination until the dura mater is opened, after local application of some drops of local anaesthetics. Propofol sedation is stopped after opening of the dura, with the

For peer review only - http://bmjopen.bmj.com/site/about/guidelines.xhtml

patient awakening with as few external stimuli as possible. Cortical stimulation is performed with a bipolar electrical stimulator. The distance between both poles is 5 mm, and stimulation is performed by placing this bipolar pincet directly on the cortical surface and stimulating with increasing electrical biphasic currents of 2–12 mA (1-2 mA increasing steps, pulse frequency 60 Hz, single pulse phase duration of 100 microsec.) until motor or speech arrest is observed. For motor mapping a 2-second train and for speech mapping a 5-second train is used, respectively. The Boston naming test and repetition of words is done in cooperation with a neuropsychologist/linguist, who will inform the neurosurgeon of any kind of speech arrest or dysarthria. The difference between these is not always clear, but can be distinguished from involuntary muscle contraction affecting speech. When localizing the motor and sensory cortex, the patient is asked to report any unintended movement or sensation in extremities or face. Confirmed functional cortical areas are marked with a number. After completion of cortical mapping, a resection of the tumour is performed as radical as possible using an ultrasonic aspirator (CUSA) and suction tube, while sparing these functional areas. When the tumour margins or white matter is encountered or when on regular neuronavigation the eloquent white matter tracts are thought to be in close proximity, subcortical stimulation (biphasic currents of 8–16 mA, 1-2 mA increasing steps, pulse frequency 60 Hz, single pulse phase duration of 100 microsec., 2-second train) is performed to localize functional tracts. If subcortical tracts are identified, resection is stopped. During the resection of the lesion close to an eloquent area, the patient is involved in a continuous dialogue with the neuropsychologist. That way the neurosurgeon has 'online'-control of these eloquent areas. In case of beginning disturbances of communication or of motor or sensory sensations the resection is cessated immediately. When, due to stimulation, an epileptic seizure occurs, this is stopped by administering some drops of iced saline on the just stimulated cortical area.. If a seizure continues, an i.v. propofol or diphantoin bolus of 0.5 mg/kg is administrated and repeated until the seizure stops. The mapping procedure is temporarily halted. If the patient is adequate, cooperative and able to carry out tasks after the seizure, the mapping procedure can continue. In the case of refractory seizures, the mapping procedure will be permanently halted and the resection will continue under general anesthesia. After resection of the tumour a final neurological examination is performed. During closure of the surgical field the patient is sedated with propofol again. After wound closure and dressing, sedation is stopped. The awake patient is transferred to the post-anaesthesia care unit (PACU), where the patient is hemodynamically and neurologically monitored for 24 hours.

(2) Asleep mapping under general anesthesia (arm 2: asleep mapping).

An IV is started on ipsilateral hand to the tumor. The patient is premedicated with up to 2 mg of midazolam. None if altered mental status (prevent further increase in ICP). Arterial (ipsilateral to tumor) catheter is inserted after induction of anesthesia. Anesthesia goals are to decrease ICP (if high), to maintain adequate CPP (at least 70 mmHg) to prevent cerebral ischemia from brain

For peer review only

retraction, and to allow intraoperative cortical motor mapping. Patients typically receive 1-2 g of cefazolin, and 4 mg of decadron before skin incision, and sometimes up to 1 g/kg of mannitol (all verified with the surgeon). The room is kept warm and patient covered as the goal is to have the core temperature above 36 C° during motor mapping. Induction with propofol. In case of increased ICP, have patient hyperventilate during preoxygenation and continue hyperventilation with mask as soon as possible after induction of anesthesia. Fentanyl up to 5 μg/kg in divided doses throughout induction, prior to intubation. Adequate neuromuscular blockade (rocuronium) is verified prior to intubation to avoid coughing/straining. Eyes are taped, and at least one additional large bore IV is inserted. Neuromuscular relaxation is let to wear off for motor mapping (do not reverse). Patient position will depend on location of tumor. Anesthesia is mainained with 70% nitrous oxide in oxygen, low dose inhalation agent (less than 0.5 MAC), and a remifentanil (0.2 μg/kg/min) or fentanyl infusion (2 μg/kg/hr). Euvolemia is maintained (Lactated Ringer's). Mild hyperventilation ($PaCO_2$ 35 mmHg) is used. Once the bone flap is removed, the surgeon assesses the tightness of the dura. ICP is further decreased if necessary (pCO_2, mannitol, propofol, head up etc.). Once the dura is open, the goal is to avoid brain shift so that stereotactic navigation system can be used optimally. During motor mapping, the arm, leg and face are uncovered to observe for movement. Stimulation is performed with the use of evoked potentials and continuous dynamic mapping/direct subcortical stimulation (CDM/DSS) with a monopolar stimulator (INOMED© Medizintechnik GmBH, Germany). During stimulation, TES-MEP registration is performed of the contralateral m. orbicularis oris, m. orbicularis oculi, m. biceps brachii, m. abductor pollicis, m. rectus femoris and m. tibialis anterior; and the ipsilateral m. abductor pollicis. SSEP registration is performed of the contralateral n. tibialis and bilateral n. medianus. The pulse form is negative, with 5 pulses and a pulse width of 500 μs, ISI 4 and current between 5-20 mA. In case of poststimulation continuation of motor activity, the surgeon will try to stop it by applying cold saline on the cortex. Have propofol (10 mg/ml) in line in case of intraoperative seizures (0.5 mg/kg for seizure suppression). May use neuromuscular relaxants after the last motor mapping. Fentanyl infusion is usually stopped at the beginning of closure. Remifentanil infusion is stopped about 10 min before end of surgery. At this point, use of inhalation agent may be replaced with a propofol infusion (50-100 μg/kg/min). pCO_2 is normalized to facilitate spontaneous breathing at the end of the operation. Use of inhalation agents (or propofol) is usually stopped about 10-15 min before end of surgery, and nitrous oxide at the end of surgery. Residual neuromuscular blockade is reversed once the Mayfield pins have been removed. At the end of the procedure all anaesthetics are stopped and patient is brought to the Post Anaesthesia Care Unit (PACU/IC). Detubation of the patient is performed as early as possible, if patient fulfils the detubation criteria (> 36 C body temperature, stable hemodynamics, sufficient spontaneous ventilation, adequate response to verbal orders). Postoperative analgesia is provided with paracetamol i.v. or p.o. 1 g up to 4 dd and

For peer review only - http://bmjopen.bmj.com/site/about/guidelines.xhtml

morphine 7.5 mg s.c. up to 4 dd, if necessary. At the post-anesthesia care unit (PACU) the patient is hemodynamically and neurologically monitored for 24 hours.

(3) Craniotomy under general anaesthesia without mapping (arm 3: no mapping).

On the evening before surgery 1.5–2.0 mg lorazepam is administered for anxiolysis. 60 min. before anaesthesia induction the patient receives 1g paracetamol p.o. and 7.5-15 mg midazolam p.o. if requested for sedation. En route to the operating room, 0.5-2 mg midazolam i.v. may be given. 1g cefazoline is given iv. for antibiotic prophylaxis before anaesthesia induction. General anaesthesia is induced intravenously with fentanyl 0.25-0.5 mg, propofol 100-200 mg and cis-atracurium 10-20 mg. After induction of anaesthesia, patient is orotracheally intubated and mechanical ventilation is applied. Respiratory rate and tidal volume are adjusted to keep the patient normocapnic.
An arterial line (alternatively: two peripheral i.v.'s), central venous catheter (v. basilica), and urinary catheter are inserted. Anaesthesia is maintained with propofol (up to 10 mg/kg/h) and remifentanil (0.5-2 µg/kg/min). isoflurane (up to 1 MAC) and clonidine (1-2 µg/kg) may be added for maintenance, if necessary (a beta blocker or calcium channel blocker may be used to control BP as an alternative to clonidine). The fluid management is aiming for normovolemia. 0.9% saline solution and balanced crystalloids are used for maintenance, in case of blood loss > 300 ml, HAES 130/0.4 solution will be given. Temperature management is aiming for normothermia, warm-air blankets and warmed infusion lines are used. Arterial blood gas analysis is performed at the beginning of the procedure and repeated, if necessary. Electrolytes are controlled and substituted and hyperglycemia will be treated with insulin, if necessary. The anesthetized patient is positioned on the table. Local infiltration of the scalp is performed with 20 ml lidocaine 1% with adrenaline 1:200.000 to reduce bleeding. The insertion points of the Mayfield clamp are not infiltrated with local anaesthetics. Trephination and tumour resection are performed without any additional neuro-psychological monitoring, guided by standard neuronavigation. At the end of the procedure all anaesthetics are stopped and patient is brought to the post-anesthesia care unit (PACU). Detubation of the patient is performed as early as possible, if patient fulfils the detubation criteria (>36 C body temperature, stable hemodynamics, sufficient spontaneous ventilation, adequate response to verbal orders). Postoperative analgesia is provided with paracetamol i.v. or p.o. 1 g up to 4 dd and morphine 7.5 mg s.c. up to 4 dd, if necessary. At the PACU the patient is hemodynamically and neurologically monitored for 24 hours.

Surgical adjuncts and additional imaging

The use of fMRI, DTI (Diffusion Tensor Imaging), ultrasound or 5-ALA is allowed to be used in all groups on the surgeon's indication.

Participant timeline

For peer review only - http://bmjopen.bmj.com/site/about/guidelines.xhtml

The flow diagram illustrates the main study procedures, including follow-up evaluations (Figure 1). In summary, study patients are allocated to either the awake mapping, asleep mapping or no mapping group and will undergo evaluation at presentation (baseline) and during the follow-up period at 6 weeks, 3 months, 6 months and 12 months postoperatively. Motor function will be evaluated using the NIHSS (National Institute of Health Stroke Scale) and MRC (Medical Research Council) scales. Language function will be evaluated using a standard neurolinguistic test-battery consisting of the Aphasia Bedside Check (ABC), Shortened Token test, Verbal fluency and Picture description. Cognitive function will be assessed using the Montreal Cognitive Assessment (MoCA). Patient functioning with be assessed with the Karnofsky Performance Scale (KPS) and the ASA (American Society of Anesthesiologists) physical status classification system. Health-related quality of life (HRQoL) will be assessed with the EQ-5D questionnaire. Overall survival and progression-free survival will be assessed at 12 months postoperatively. We expect to complete patient inclusion in 4 years. The estimated duration of the study (including follow-up) will be 5 years.

<u>Study procedures: Clinical evaluations and follow-up</u>

- Pre-op (baseline) CRF
  - Unique subject ID, demographics (centre, year, gender, age), tumor specific factors (tumor volume pre-op, tumor hemisphere and lobe; eloquent areas), patient specific factors: preoperative KPS, ASA score, neurological status (NIHSS), MRC grade arm/leg, neurolinguistic testing, MOCA, EQ-5D.

- Surgery CRF
  - Type of ISM, surgeon's rationale for modality, use of surgical adjuncts, use of additional imaging, radiological factors: resection percentage (both the contrast-enhancing and non-contrast-enhancing part), residual volume and postoperative ischemia.

- Follow-up CRFs
  - 6 weeks postoperatively: histology and molecular markers (WHO grade, MGMT status, IDH-1 status), neurological status (NIHSS), MRC grade arm/leg, status MRC arm/MRC leg/facialis/speech/visual (new, worsened, improved, stable), KPS, MOCA, EQ-5D.
  - 3 months postoperatively: neurological status (NIHSS), MRC grade arm/leg, status MRC arm/MRC leg/facialis/speech/visual (new, worsened, improved, stable), KPS, neurolinguistic testing, MOCA, EQ-5D.

For peer review only

- 6 months postoperatively: neurological status (NIHSS), adjuvant treatment, MRC grade arm/leg, status MRC arm/MRC leg/facialis/speech/visual (new, worsened, improved, stable), KPS, MOCA, EQ-5D.
- 12 months postoperatively: progression-free survival, overall survival (end-of-study).

Outcomes

Primary outcome measures

The primary outcomes are 1) proportion of patients with NIHSS (National Institute of Health Stroke Scale) deterioration at 6 weeks, 3 months and 6 months postoperatively; deterioration is defined as an increase of at least one point on the total NIHSS score compared to this score at baseline and 2) residual tumor volume of the contrast-enhancing and non-contrast enhancing part, as assessed by a neuroradiologist on postoperative T1 with contrast MRI scan sequences using manual or semi-automatic volumetric analyses (Brainlab Elements iPlan CMF Segmentation, Brainlab AG, Munich, Germany; or similar software).

Secondary outcome measures

The secondary outcomes are 1) progression-free survival (PFS) at 12 months defined as time from diagnosis to disease progression (occurrence of a new tumor lesions with a volume greater than 0.175 cm^3, or an increase in residual tumor volume of more than 25%) or death, whichever comes first; 2) overall survival (OS) at 12 months defined as time from diagnosis to death from any cause; 3) onco-functional outcome defined as the calculated coordinate of the EOR on the x-axis and the postoperative NIHSS deterioration on the y-axis and 4) frequency and severity of Serious Adverse Events (SAEs) in each arm.

NIHSS

The National Institutes of Health Stroke Scale, or NIH Stroke Scale (NIHSS) is a tool used by healthcare providers to objectively quantify the impairment caused by a stroke, but has been used extensively for outcome in glioma surgery because of the lack of such scale for neuro-oncologic purposes and has been validated. The NIHSS is composed of 11 items, each of which scores a specific ability between a 0 and 4. For each item, a score of 0 typically indicates normal function in that specific ability, while a higher score is indicative of some level of impairment. The individual scores from each item are summed in order to calculate a patient's total NIHSS score. The maximum possible score is 42 and the minimum score 0.

Aphasia Bedside Check (ABC)

For peer review only - http://bmjopen.bmj.com/site/about/guidelines.xhtml

ABC is a short screening test to detect aphasic disturbances at language comprehension and language production level at the main linguistic levels. It consists of 14 items in total. The cut-off score for signs of aphasia is ≤12.

Shortened Token Test

The shortened Token Test is a test for language comprehension and for the severity of a language disorder. The patient is asked to point and to manipulate geometric forms on verbal commands. It consists of 36 items. The cut-off score is 29.5.

Verbal fluency (category and letter)

Category and letter fluency are tests to assess flexibility of verbal semantic and phonological thought processing, semantic memory and concept generation. The patients is asked to produce words of a given category (animals, professions) or beginning with a given letter (D, A, T) within a limited time span.

Picture description

This is a subtest from the CAT-NL to assess semi-spontaneous speech in an oral and written way (5 minutes each condition). Scoring can be done according to the manual or more thoroughly according to the variables mentioned by Vandenborre et al[20].

Montreal Cognitive Assessment (MOCA)

The MOCA is a cognitive screening test to detect mild impairments across several cognitive domains; attention, verbal memory, language, visuo-constructive skills, conceptual thought, calculation and orientation. The total score is 30, the cut-off score is ≤26.

EQ-5D

The EQ-5D is a standardized questionnaire to assess the general health-related quality of life (HRQoL) in five domains: mobility, self-care, usual activity, pain/discomfort and anxiety/depression. It is developed by the EuroQol Group and can also be used to calculate quality-adjusted life years (QALYs) for cost-utility analyses.

Sample size

This study has two primary endpoints. In order to guarantee that the overall type I error rate does not exceed 5%, we apply a weighted Bonferroni correction for multiple testing. The sample size calculations that follow take that into account. For the first primary endpoint, proportion of patients with neurological deterioration at 6 weeks post- surgery, we assume a deterioration rate of 10% in the control group (arm 3: no mapping), and 3% in the experimental groups (arm 1 and 2: awake and

For peer review only

asleep mapping). A two-sample test for proportions with continuity correction requires 411 patients (137 per arm) in total in order to detect the above-mentioned difference of 7% with 80% power at a 4% significance level. For the second primary endpoint, proportion of patients without residual contrast-enhancing tumor on postoperative MRI, we assume a success rate of 25% in the control group (arm 3: no mapping), and 50% in the experimental groups (arm 1 and 2: awake and asleep mapping). A two-sample test for proportions with continuity correction requires 188 patients (94 per arm) in total in order to detect the above-mentioned difference of 25% with 80% power at a 1% significance level. In order to power the study for both primary endpoints, we should include the larger required number of patients, i.e. 411. A total of 411 eligible and evaluable patients in three arms allow the difference of 25% in proportion of patients without residual tumor to be detected with 88% power. Taking into account possible ineligibility and withdrawal of consent (we estimate this at 10%), a total of 453 patients will be included (151 patients per arm).

Data collection

All patient data is collected in the electronic data software Castor EDC. This software allows built-in logical checks and validations to promote data quality. Data entry and group allocation is performed by the study coordinator or locally by trained physicians and research nurses under supervision of the local investigator.

Data analysis

All analyses will be according the intention to treat principle, restricted to eligible patients. Patients initially registered but considered ineligible afterwards based on the histological analysis on tissue extracted during surgery, will be excluded from all analyses.

Primary study parameters

The primary endpoints will be analyzed using multivariate logistic regression. Subgroup analyses for tumor grade (WHO grade III/IV), preoperative neurological morbidity, preoperative tumor volume, patient's age (in 10-year age brackets) and tumor location/eloquence will be performed.

We will be including a stratification factor in the primary analysis model with each 10 observed events using the order of prognostic value as mentioned in the paragraph above, where the first 10 events will be used to estimate the effect of the arm. This rule will be applied in case less than 40 patients in total develop neurological deterioration. In the so constructed multivariate logistic regression model the treatment arm effect will be tested at 4% significance level. The primary analysis of proportion of patients without residual contrast-enhancing and non-contrast-enhancing tumour consist of a multivariate logistic regression, where arm effect is corrected for all minimization factors. In this model the group effect will be tested at 1% significance level. Manual or semiautomatic segmentation will be performed on axial T1 MRI contrast enhanced slices to measure

For peer review only

preoperative and postoperative tumor volume. A determination of volumes will be calculated blinded for the treatment group.

Secondary study parameters

The Kaplan-Meier method will be used to estimate PFS and OS proportions per treatment group at appropriate time points, while the Greenwood estimate of the standard error will be used to construct the corresponding 95% CI. Multivariate cox proportional hazards models will be built for PFS and OS where treatment group effect will be corrected for minimization factors age group (≤55 years vs >55years), KPS (80–90 vs >90), and left or right hemisphere. Additionally, competing risk analysis will be used to calculate cumulative incidence of PFS (with competing risks progression/relapse and death without progression/relapse which add up to 100% at every time point). Onco-functional outcome will be evaluated using a scatter or bubble plot with volumetric data on the x-axis and neurological status (NIHSS) or patient performance (KPS) on the y-axis. SAEs in both groups will be described.

Study monitoring

No scheduled on-site monitoring visits will be performed. Local investigators will remain responsible for the fact that the rights and well-being of patients are protected, the reported trial data are accurate, complete, and verifiable from source documents and the conduct of the trial is in compliance with the currently approved protocol/amendment(s), with GCP, and with the applicable regulatory requirement(s). Direct access to source documentation (medical records) must be allowed for the purpose of verifying that the data recorded in the CRF are consistent with the original source data. No Data Safety Monitoring Board will be installed: all interventions are care-as-usual and patients are allocated without randomisation.

Adverse events (AEs) and serious adverse events (SAEs)

Adverse events are defined as any undesirable experience occurring to a subject during the study, whether or not considered related to neurosurgery. All adverse events reported spontaneously by the subject or observed by the investigator or his staff will be recorded from start of surgery until 6 weeks after surgery. Serious adverse events are any untoward medical occurrence or effect that results in death; is life-threatening (at the time of the event); requires hospitalization or prolongation of existing inpatients' hospitalization; results in persistent or significant disability or incapacity or any other important medical event that did not result in any of the outcomes listed above due to medical or surgical intervention, but could have been based upon appropriate judgement by the investigator. An elective hospital admission will not be considered as a serious adverse event. Most of the (serious) adverse effects of treatments be mainly related to the surgery: post operative pain, nausea and

For peer review only

anaemia (in case of massive blood loss), Infections, intracranial haemorrhage, epilepsy, aphasia, paresis/paralysis in arms or/and legs.

Most of the (serious) adverse effects of treatments (awake surgery or surgery under generalised anaesthesia) will be mainly related to the surgery: postoperative pain, nausea and anaemia (in case of massive blood loss), infections, intracranial haemorrhage, epilepsy, aphasia, paresis/paralysis in arms or/and legs. The neurological morbidity is under investigation in this trial and well-known risk / complications of the craniotomy and can be attributed to the nature of the operation. Neurosurgical clinics are well adapted to prevent and treat such events. SAEs will be collected through routine data management.

Publication of results

Trial results will be published in an international journal, communicated to neurological and neurosurgical associations and presented at (inter)national congresses.

Ethics and Dissemination

The study has been approved by the Medical Ethics Committee (METC Zuid-West Holland/Erasmus Medical Center; MEC-2020-0812) and is conducted in complicance with the European Union Clinical Trials Directive (2001/20/EC) and the principles of the Declaration of Helsinki (2013). The results of the study will be published in peer-reviewed academic journals and disseminated to patient organisations and media.

For peer review only

DISCUSSION

Neurosurgeons face a major dilemma during glioma surgery: maximizing extent of resection while minimizing risk of postoperative neurological deficits. The use of awake or asleep mapping techniques has the potential to equip the surgeon intraoperatively with the needed information to balance these two surgical goals.

A substantial amount of evidence is available on the usefulness of awake mapping to increase resection percentage while preserving quality of life in low-grade glioma patients[21-32]. In contrast, only very few studies have reported the use of awake mapping in high-grade glioma patients, although this technique could be of important value in these patients as well[17,21, 23-25,32]. Recent retrospective evidence showed that glioblastoma patients operated with awake mapping had significant less postoperative neurological morbidity and significantly higher percentage of total resections[33,34]. In patients with motor-eloquent tumors, the use of asleep mapping techniques with evoked potentials or continuous dynamic mapping can be a viable alternative to preserve these functional tracts[35-37].

There is a clear need for solid prospective evidence of the use of these techniques in HGG patients. The presented international neurosurgical research consortium will provide the needed infrastructure to perform ongoing large-scale data collection[18]. This study aims to evaluate whether the use of awake or asleep mapping is the appropriate answer to the surgeon's surgical dilemma during high-grade glioma resections. Furthermore, it will be the first to directly compare awake and asleep mapping techniques in their ability to improve patient outcomes for neurological morbidity, quality of life and survival. Last, using various multivariate analyses, there will be an additional focus on identifying the best surgical choice in subgroups of high-grade glioma patients.

<u>Trial status</u>

The study will start at April 1st, 2021 and is open to additional participating neurosurgical centers.

For peer review only - http://bmjopen.bmj.com/site/about/guidelines.xhtml

Figure 1: Study flow diagram

ABBREVIATIONS

5-ALA: 5-Aminolevulinic Acid

AC: Awake Craniotomy

AE: Adverse Event

CI: Confidence Interval

CTC: Clinical Trial Center

DTI: Diffusion Tensor Imaging

EMC: Erasmus Medical Center

EOR: Extent of Resection

fMRI: Functional Magnetic Resonance Imaging

GA: General Anesthesia

GCP: Good Clinical Practice

GTR: Gross-Total Resection

HMC: Haaglanden Medical Center

HRQoL: Health-related Quality of Life

ISM: Intraoperative Stimulation Mapping

IRB: Institutional Review Board

KPS: Karnofsky Performance Score

LGG: Low-grade glioma

METC: Medical Ethics Committee

MOCA: Montreal Cognitive Assessment

NIHSS: National Institute of Health Stroke Scale

OS: Overall survival

PACU: Post-Anesthesia Care Unit

PFS: Progression-free survival

SAE: Serious Adverse Event

STR: Subtotal Resection

UCSF: Univeristy of California, San Francisco

WHO: World Health Organization

For peer review only

DECLARATIONS

Author contributions

JG, AV and MLB designed the study, wrote the study protocol and are end-responsible for the implementation and organization of the study in all participating centers. JG wrote the study protocol and is responsible for the implementation and organization of the study in all participating centers and the conduct of the database. CD contributed to the design of the study. SV contributed to the design of the study and is responsible for the local conduct of the study in Leuven. PS contributed to the design of the study and is responsible for the local conduct of the study in Bern. CJ contributed to the design of the study and is responsible for the local conduct of the study in Heidelberg. SK contributed to the design of the study and is responsible for the local conduct of the study in Munich. BN contributed to the design of the study and is responsible for the local conduct of the study in Boston. MSB contributed to the design of the study and is responsible for the local conduct of the study in San Francisco. MLB contributed to the design of the study and is responsible for the local conduct of the study in The Hague. All authors read and approved the final version of the manuscript.

Ethics approval and consent to participate

The study has been approved Medical Ethics Committee (IRB/METC Zuid-West Holland/Erasmus Medical Center; MEC-2020-0812) and is conducted in complicance with the European Union Clinical Trials Directive (2001/20/EC) and the principles of the Declaration of Helsinki (2013).

Consent for publication

By giving written informed consent, patients agree with the storage of data and publication of the study results.

Competing interests

The authors declare that they have no competing interests.

For peer review only

For peer review only - http://bmjopen.bmj.com/site/about/guidelines.xhtml

REFERENCES

1. DeAngelis LM (2001) Brain tumors. *N Engl J Med.* 344:114-123
2. Omuro A, DeAngelis LM (2013) Glioblastoma and other malignant gliomas: A clinical review. *JAMA.* 310:1842-1850
3. Stupp R, Mason WP, van den Bent MJ, Weller M, Fisher B, Taphoorn MJ, Belanger K, Brandes AA, Marosi C, Bogdahn U, Curschmann J, Janzer RC, Ludwin SK, Gorlia T, Allgeier A, Lacombe D, Cairncross JG, Eisenhauer E, Mirimanoff RO (2005) Radiotherapy plus concomitant and adjuvant temozolomide for glioblastoma. *N Engl J Med.* 352:987-996
4. Ho VK, Reijneveld JC, Enting RH, Bienfait HP, Baumert BG, Visser O (2014) Changing incidence and improved survival of gliomas. *Eur J Cancer.* 50:2309-2318
5. Eckel-Passow J.E. et al. Glioma groups based on 1p/19q, IDH, and TERT promotor mutations in tumors. *N Engl J Med.* 2015. 372:2499-508
6. Taal W, Oosterkamp HM, Walenkamp AM et al. Single-agent bevacizumab or lomustine versus a combination of bevacizumab plus lomustine in patients with recurrent glioblastoma (BELOB trial): a randomized controlled phase 2 trial. *Lancet Oncol.* 2014.15:943-53
7. Stummer W, Pichlmeier U, Meinel T et al. Fluorescence-guided surgery with 5-aminolevulinic acid for resection of malignant glioma: randomized controlled multicentre phase III trial. *Lancet Oncol.* 2006. 7:392-401
8. Smith JS, Cha S, Chang EF, Chang SM, Lamborn KR, Prados MD, Cha S, Tihan T, Vandenberg S, McDermott MW, Berger MS (2008) Role of extent of resection in the long-term outcome of low-grade hemispheric gliomas. *J Clin Oncol.* 26:1338-1345
9. Stummer W, Meinel T, Pichlmeier U, Reulen HJ, Schumacher W, Tonn JC, Rohde V, Oppel F, Turowski B, Woiciechoswky C, Franz K, Pietsch T (2008) Extent of resection and survival in glioblastoma multiforme: Identification of and adjustment for bias. *Neurosurgery.* 62:564-576
10. Sanai N, Berger MS (2008) Glioma extent of resection and its impact on patient outcome. *Neurosurgery*. 62:753-764
11. Vourinen V, Hinkka S, Färkkilä M, Jääskeläinen J, (2003) Debulking or biopsy of malignant glioma in elderly people – randomized study. *Acta Neurochir.* 145:5-10.
12. Lacroix M, Abi-Said S, Fourney DR, Gokaslan ZL, Shi W, DeMonte F, Lang FF, McCutcheon IE, Hassenbusch SJ, Holland E, Hess K, Michael C, Miller D, Sawaya R (2001) A multivariate analysis of 416 patients with glioblastoma multiforme: prognosis, extent of resection, and survival. *J Neurosurg.* 95:190-198
13. Molinaro AM, Hervey-Jumper S, Morshed RA et al (2020) Association of maximal extent of resection of contrast-enhanced and non-contrast-enhanced tumor with survival within molecular subgroups of patients with newly diagnosed glioblastoma. *JAMA Oncol.* 6:495-503.

For peer review only - http://bmjopen.bmj.com/site/about/guidelines.xhtml

14. Keles GE, Anderson B, Berger MS (1999) The effect of extent of resection on time to tumor progression and survival in patients with glioblastoma multiforme of the cerebral hemisphere. *Surg Neurol.* 52:371-379

15. Keles GE, Lamborn KR, Chang SM, Prados MD, Berger MS (2004) Volume of residual disease as a predictor of outcome in adult patients with recurrent supratentorial glioblastomas multiforme who are undergoing chemotherapy. *J Neurosurg.* 100:41-46

16. Van den Bent MJ, Stupp R, Mason W (2005) Impact of the extent of resection on overall survival in newly diagnosed glioblastoma after chemo-irradiation with temozolomide: further analyses of EORTC study 26981. *Eur J Cancer Suppl.* 3:134

17. De Witt Hamer PC, Robles SG, Zwinderman AH, Duffau H, Berger MS (2012) Impact of intraoperative stimulation brain mapping on glioma surgery outcome: a meta-analysis. *J Clin Oncol.* 30:2559-2565

18. Gerritsen JKW, Broekman MLD, de Vleeschouwer S et al (2021) Letter: The European and North American Consortium and Registry for intraoperative stimulation mapping: framework for a transatlantic collaborative research initiative. *Neurosurgery.* 88:E369. doi: 10.1093/neuros/nyaa568

19. Sawaya R, Hammoud M, Schoppa D et al. Neurosurgical outcomes in a modern series of 400 craniotomies for treatment of parenchymal tumors. *Neurosurgery.* 1998. 42:1044-55

20. Vandenborre D, Visch-Brink E, van Dun K et al. Oral and written picture description in individuals with aphasia. *Int J Lang Commun Disord.* 2018. 53:294-307

21. Gupta DK, Chandra PS, Ohja BK, Sharma BS, Mahapatra AK, Mehta VS (2007) Awake craniotomy versus surgery under general anesthesia for resection of intrinsic lesions of eloquent cortex – a prospective randomized study. *Clin Neurol Neurosurg.* 109:335-343

22. Brown TJ, Brandmeir NJ, Church EW, Brennan MC, Li M, Rakszawski KL, Patel AS, Rizk EB, Suki D, Sawaya R, Glantz M (2016) Association of the extent of resection with survival in glioblastoma: A systematic review and meta-analysis. *JAMA Oncol.* 2:1460-1469

23. Peruzzi P, Bergese SD, Viloria A, Puente EG, Abdel-Rasoul M, Chiocca EA (2011) A retrospective cohort-matched comparison of conscious sedation versus general anesthesia for supratentorial glioma resection. Clinical article. *J Neurosurg.* 114:633-639

24. Sacko O, Lauwers-Cances V, Brauge D, Sesay M, Brenner A, Roux FE (2011) Awake craniotomy vs surgery under general anesthesia for resection of supratentorial lesions. *Neurosurgery.* 68:1992-1998

25. Hervey-Jumper SL, Li J, Lau D, Molinaro AM, Perry DW, Meng L, Berger MS (2015) Awake craniotomy to maximize glioma resection: methods and technical nuances over a 27-year period. *J Neurosurg.* 123:325-339

For peer review only

26. Danks RA, Aglio LS, Gugino LD, Black PM (2000) Craniotomy under local anesthesia and monitored conscious sedation for the resection of tumors involving eloquent cortex. *J Neurooncol* 49:131-139
27. De Benedictis A, Moritz-Gasser S, Duffau H (2010) Awake mapping optimizes the extent of resection for low-grade gliomas in eloquent areas. *Neurosurgery.* 66:1074-1084
28. Duffau H (2009) A personal consecutive series of surgically treated 51 cases of insular WHO Grade II glioma: advances and limitations. *J Neurosurg.* 110:696-708
29. Duffau H, Capelle L, Denvil D, Sichez N, Gatignol P, Lopes M, Mitchell MC, Sichez JP, Van Effenterre R (2003) Functional recovery after surgical resection of low grade gliomas in eloquent brain: hypothesis of brain compensation. *J Neurol Neurosurg Psychiatry*. 74:901-907
30. Duffau H, Capelle L, Denvil D, Sichez N, Gatignol P, Taillandier L, Lopes M, Mitchell MC, Roche S, Muller JC, Bitar A, Sichez JP, van Effenterre R (2003) Usefulness of intraoperative electrical subcortical mapping during surgery for low-grade gliomas located within eloquent brain regions: functional results in a consecutive series of 103 patients. *J Neurosurg.* 98:764-778
31. Duffau H, Lopes M, Arthuis F, Bitar A, Sichez JP, Van Effenterre R, Capelle L (2005) Contribution of intraoperative electrical stimulations in surgery of low grade gliomas: a comparative study between two series without (1985-96) and with (1996-2003) functional mapping in the same institution. *J Neurol Neurosurg Psychiatry.* 76:845-851
32. Yoshikawa K, Kajiwara K, Morioka J, Fuji M, Tanaka N, Fujisawa H, Kato S, Nomura And S, Suzuki M (2006) Improvement of functional outcome after radical surgery in glioblastoma patients: the efficacy of navigation-guided fence-post procedure and neurophysiological monitoring. *J Neurooncol.* 78:91-97
33. Gerritsen JK, Arends LR, Dirven CM, Schouten JW, Klimek M, Vincent AJ (2017) Impact of intraoperative stimulation mapping and awake craniotomy on high-grade glioma surgery outcome: A meta-analysis. *Acta Neurochirurgica (Wien).* 161:99-107
34. Gerritsen JK, Viëtor CL, Rizopoulos D, Schouten JW, Klimek M, Dirven CM, Vincent AJ (2017) Awake craniotomy versus craniotomy under general anesthesia for supratentorial high-grade glioma in eloquent areas: A retrospective controlled-matched study. *Acta Neurochirurgica (Wien).* 161:307-15
35. Krieg SM, Shiban E, Droese D et al. Predictive value and safety of intraoperative neurophysiological monitoring with motor evoked potentials in glioma surgery. *Neurosurgery.* 2012. 70:1060-70
36. Raabe A, Beck J, Schucht P et al. Continuous dynamic mapping of the corticospinal tract during surgery of motor eloquent brain tumors: evaluation of a new method. *J Neurosurg.* 2014. 120:1015-24

For peer review only

For peer review only - http://bmjopen.bmj.com/site/about/guidelines.xhtml

37. Schucht P, Seidel K, Jilch A et al. A review of monopolar motor mapping and a comprehensive guide to continuous dynamic motor mapping for resection of motor eloquent brain tumors. *Neurochirurgie.* 2017. 63:175-80

For peer review only

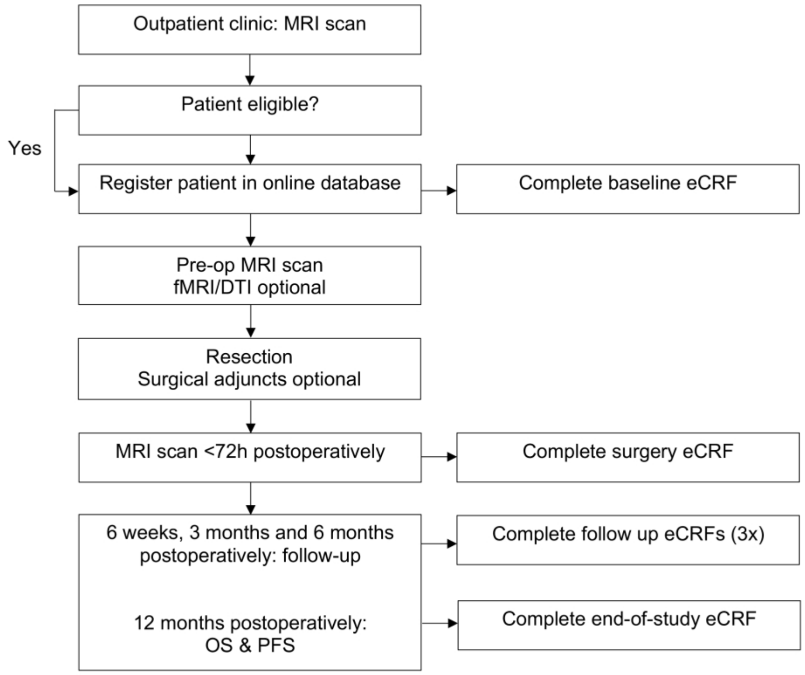

Figure 1: Study flowchart

209x297mm (300 x 300 DPI)

STROBE Statement—Checklist of items that should be included in reports of ***cohort studies***

	Item No	Recommendation	Page No
**Title and abstract**	1	(*a*) Indicate the study's design with a commonly used term in the title or the abstract	1,2
		(*b*) Provide in the abstract an informative and balanced summary of what was done and what was found	1,2
**Introduction**			
Background/rationale	2	Explain the scientific background and rationale for the investigation being reported	3,4
Objectives	3	State specific objectives, including any prespecified hypotheses	3,4
**Methods**			
Study design	4	Present key elements of study design early in the paper	5
Setting	5	Describe the setting, locations, and relevant dates, including periods of recruitment, exposure, follow-up, and data collection	5
Participants	6	(*a*) Give the eligibility criteria, and the sources and methods of selection of participants. Describe methods of follow-up	5,6
		(*b*) For matched studies, give matching criteria and number of exposed and unexposed	
Variables	7	Clearly define all outcomes, exposures, predictors, potential confounders, and effect modifiers. Give diagnostic criteria, if applicable	5
Data sources/ measurement	8*	For each variable of interest, give sources of data and details of methods of assessment (measurement). Describe comparability of assessment methods if there is more than one group	5,10,11
Bias	9	Describe any efforts to address potential sources of bias	12
Study size	10	Explain how the study size was arrived at	12,13
Quantitative variables	11	Explain how quantitative variables were handled in the analyses. If applicable, describe which groupings were chosen and why	11-13
Statistical methods	12	(*a*) Describe all statistical methods, including those used to control for confounding	13,14
		(*b*) Describe any methods used to examine subgroups and interactions	
		(*c*) Explain how missing data were addressed	
		(*d*) If applicable, explain how loss to follow-up was addressed	
		(*e*) Describe any sensitivity analyses	
**Results**			
Participants	13*	(a) Report numbers of individuals at each stage of study—eg numbers potentially eligible, examined for eligibility, confirmed eligible, included in the study, completing follow-up, and analysed	NA
		(b) Give reasons for non-participation at each stage	
		(c) Consider use of a flow diagram	
Descriptive data	14*	(a) Give characteristics of study participants (eg demographic, clinical, social) and information on exposures and potential confounders	NA
		(b) Indicate number of participants with missing data for each variable of interest	
		(c) Summarise follow-up time (eg, average and total amount)	
Outcome data	15*	Report numbers of outcome events or summary measures over time	NA

For peer review only

Main results	16	(*a*) Give unadjusted estimates and, if applicable, confounder-adjusted estimates and their precision (eg, 95% confidence interval). Make clear which confounders were adjusted for and why they were included<br>(*b*) Report category boundaries when continuous variables were categorized<br>(*c*) If relevant, consider translating estimates of relative risk into absolute risk for a meaningful time period	NA
Other analyses	17	Report other analyses done—eg analyses of subgroups and interactions, and sensitivity analyses	NA
**Discussion**			
Key results	18	Summarise key results with reference to study objectives	NA
Limitations	19	Discuss limitations of the study, taking into account sources of potential bias or imprecision. Discuss both direction and magnitude of any potential bias	16,17
Interpretation	20	Give a cautious overall interpretation of results considering objectives, limitations, multiplicity of analyses, results from similar studies, and other relevant evidence	16,17
Generalisability	21	Discuss the generalisability (external validity) of the study results	16,17
**Other information**			
Funding	22	Give the source of funding and the role of the funders for the present study and, if applicable, for the original study on which the present article is based	1

*Give information separately for exposed and unexposed groups.

Note: An Explanation and Elaboration article discusses each checklist item and gives methodological background and published examples of transparent reporting. The STROBE checklist is best used in conjunction with this article (freely available on the Web sites of PLoS Medicine at http://www.plosmedicine.org/, Annals of Internal Medicine at http://www.annals.org/, and Epidemiology at http://www.epidem.com/). Information on the STROBE Initiative is available at http://www.strobe-statement.org.

For peer review only
